# Continued post-weaning social isolation is associated with persistent, sex-specific changes in sociability and social interaction dynamics in mice

**DOI:** 10.1371/journal.pone.0361386

**Published:** 2026-10-26

**Authors:** Kazuhiko Yamamuro, Masato Takahashi, Mamiko Okuda, Kazuki Okumura, Natsuko Kashida, Takahiro Yoshihara, Tsutomu Takeda, Michihiro Toritsuka, Takahira Yamauchi, Manabu Makinodan

**Affiliations:** 1 Center for Health Control, Nara Medical University, Kashihara, Nara, Japan; 2 Department of Psychiatry, School of Medicine, Nara Medical University, Kashihara, Nara, Japan; 3 Department of Neuropsychiatry, Kumamoto University School of Medicine, Kumamoto, Kumamoto, Japan; Ohio State University, UNITED STATES OF AMERICA

## Abstract

Early social experience is critical for the maturation of social behavior, and its disruption during juvenile development may confer long-term psychiatric vulnerability. Juvenile social isolation (j-SI) after weaning is widely used as a model of early social deprivation; however, its developmental and sex-specific effects on spontaneous social interaction remain unclear. We examined the behavioral consequences of post-weaning social isolation in male and female C57BL/6J mice (*Mus musculus*). Mice were individually housed from postnatal day (P)21, and spontaneous social interaction was assessed longitudinally at P35, P49, and P63 during prolonged cage-based sessions using an augmented reality-based behavioral monitoring system. Locomotor activity, inter-mouse distance, mean contact duration per initiated encounter, and interaction dynamics were quantified during the light and dark phases. Juvenile social isolation was associated with greater inter-mouse distance across most developmental stages and sex-phase combinations, whereas reductions in mean contact duration per initiated encounter varied by sex, developmental stage, and light/dark phase. Locomotor activity varied by developmental stage and sex, with late-emerging increases particularly evident during the dark phase in isolated mice. In contrast, lower interaction-partner switching in isolated males was detected only at the earliest stage (P35) during the light phase. An important design feature is that isolated mice were reunited at testing with cage mates from which they had been separated for more than two weeks and were therefore effectively unfamiliar, whereas group-housed mice were tested with continuously familiar cage mates; the reported effects therefore reflect social isolation together with its intrinsic reduction in social familiarity. These findings indicate that continued post-weaning social isolation is associated with persistent, sex-specific alterations in sociability and social interaction dynamics. Our results further suggest that juvenile social experience may shape the developmental maturation and temporal organization of social interaction across adolescence and early adulthood.

## Introduction

Early social experiences are essential for typical neurobehavioral development across species, and disruption during early life can lead to persistent social and emotional impairments. For example, infant primates raised in social isolation develop severe behavioral abnormalities [1], and puppies isolated from humans beyond a critical period fail to socialize normally [2]. In humans, childhood neglect and institutional rearing are associated with enduring cognitive, emotional, and social impairments [3], including executive-function impairment [4] and increased anxiety and depression later in life [5]. In addition, neuroimaging studies have associated early social deprivation with altered brain development, including reduced prefrontal–temporal metabolism and abnormal white matter connectivity, measured by positron emission tomography and diffusion tensor imaging, respectively [6].

Animal models have helped clarify these behavioral effects and their underlying mechanisms. In rodents, the juvenile social isolation (j-SI) paradigm—which involves individual housing immediately after weaning, typically from postnatal day (P)21 to P35 [7–11]—is widely used to examine how disrupted early social experience affects neurobehavioral development. The timing of social isolation appears critical: isolation immediately after weaning produces enduring effects, whereas similar isolation during later adolescence or adulthood has weaker effects [7–9]. Together, these findings indicate that P21–P35 is a sensitive period for social experience-dependent behavioral maturation in mice.

Adolescence is a critical developmental period characterized by substantial reorganization of social behavior and increased sensitivity to social experiences [12,13]. During this stage, humans and rodents exhibit marked changes in social reward processing, peer interactions, and sex-specific behavioral trajectories. In humans, social disconnection and loneliness during early adolescence have been associated with lasting changes in emotional and social functioning [14,15]. Similarly, post-weaning social isolation in rodents disrupts the maturation of social circuits and leads to persistent changes in social behavior [10,11,16–18]. Specifically, post-weaning isolation has been associated with increased aggression toward conspecifics [19,20] and, in social-preference paradigms, increased social preference or hypersocial behavior in males and females [21,22]; conversely, young-adult females have shown reduced social preference after post-weaning isolation [22]. These apparently divergent findings may partly reflect assay differences, because short-term preference-based tests and long-term naturalistic paradigms can yield markedly different outcomes [19,23,24]. Together, these findings support adolescence as a sensitive window in which social experience shapes long-term behavioral outcomes.

At the neural level, j-SI produces long-lasting alterations in brain regions governing higher-order cognition, particularly the prefrontal cortex (PFC) [7–11,13–17]. Several studies have shown that post-weaning social isolation causes persistent reductions in myelination and oligodendrocyte maturation in the medial PFC, accompanied by enduring deficits in PFC-dependent cognition [10,11], as well as sex-specific alterations in dendritic spine density in the PFC and nucleus accumbens [25]. j-SI also disrupts synaptic and microcircuit functions within the PFC, leading to imbalances between excitatory and inhibitory transmission [9,10,14]. Additionally, j-SI affects the mesolimbic dopamine and stress-regulatory systems, enhancing reward-related plasticity and altering hypothalamic–pituitary–adrenal axis responsivity [18]. Evidence also indicates that the effects of j-SI differ by sex. Historically, most preclinical studies of stress-related behaviors have predominantly used male animals, largely to avoid variability associated with the female estrous cycle. However, studies directly comparing males and females indicate that stress-related vulnerabilities differ by sex, consistent with established sex differences in human stress-related psychiatric disorders [19]. Males often show greater susceptibility to isolation-induced cognitive impairment, depressive-like behavior, and stress dysregulation, whereas females exhibit relative resilience in several behavioral domains [26]; conversely, females may show heightened neural sensitivity to acute social separation, with rapid activation of hypothalamic stress circuits [27]. Long-term behavioral consequences may also differ by sex, with reports of increased sociability in isolated males but reduced social preference in females [21,22].

Previous studies have extensively examined the effects of social isolation on mouse social behavior, with a particular focus on male aggression and post-weaning isolation paradigms [19,26]. However, many have relied on short-term behavioral assays involving dyadic interactions or a single social stimulus [23,24], which capture only selected aspects of social behavior. Consequently, how social isolation affects the spontaneous organization of social behavior across development in both sexes during prolonged group-based observation remains unclear. Recent advances in automated behavioral tracking have enabled continuous monitoring of group interactions [27–30], supporting a more comprehensive assessment of social behavior.

We therefore investigated how post-weaning juvenile social isolation influences the developmental maturation of spontaneous social behavior in male and female mice. We examined social behavior longitudinally across adolescence and early adulthood with sex treated as a biological variable. This longitudinal approach may clarify how adverse social experiences shape long-term trajectories of social behavior.

## Materials and methods

### Mice and housing conditions

All experimental procedures were approved by the Institutional Animal Care and Use Committee of Nara Medical University (Approval No. 13588) and were conducted in accordance with its guidelines. C57BL/6J mice were purchased from CLEA Japan, Inc. (Tokyo, Japan). j-SI was initiated immediately after weaning at postnatal day (P)21 by housing mice individually and was maintained throughout the study period. Control mice were group-housed (GH), with four littermates per cage from weaning onward. Group sizes were as follows: male GH, n = 32 (8 four-mouse cages); male j-SI, n = 20 (5 four-mouse test groups); female GH, n = 24 (6 four-mouse cages); and female j-SI, n = 20 (5 four-mouse test groups), yielding 24 independent four-mouse groups. Behavior was assessed longitudinally in 15-h sessions beginning during the light phase on P35, P49, and P63 and continuing through the subsequent dark phase into P36, P50, and P64, respectively; throughout the manuscript, P35, P49, and P63 denote the session starting days. Mice assigned to j-SI remained individually housed throughout the experimental period and were temporarily reunited during behavioral testing with their original littermates, with whom they had been co-housed only before weaning (until P21). Because these mice had then been separated for more than two weeks before the first test (P35) and for progressively longer before the later tests (P49 and P63)—intervals that exceed the typical duration of rodent social recognition memory—the reunited partners were effectively unfamiliar at testing. In contrast, group-housed mice were tested with cage mates with whom they had remained continuously familiar. This difference in social familiarity is therefore inherent to the isolation manipulation and cannot be separated from it in the present design (see Limitations). After each testing session, j-SI mice returned to individual housing. All mice were maintained under a 12:12-h light/dark cycle (lights on at 08:00 and off at 20:00) under controlled temperature and humidity, with food and water available ad libitum. All procedures were designed to minimize the number of animals used and any pain or distress. Attachment of the identification marker was the only invasive procedure and was performed under isoflurane anesthesia (1.5–2%); because no surgery was performed, analgesics were not administered. Animals were monitored daily, and no humane endpoint was reached during the study. At the end of the experimental period, mice were euthanized by cervical dislocation under deep isoflurane anesthesia, in accordance with the AVMA Guidelines for the Euthanasia of Animals. Because the study involved only animals, informed consent was not applicable.

### Behavioral testing: Augmented reality-based long-term animal behavior observing system

We used an augmented reality-based long-term animal behavior observing (AR-LABO) system to track and quantify social interaction (Fig 1A). At least 1 week before testing, each mouse was fitted with an identification (ID) tag bearing a printed ArUco marker on its back; the tag was attached under anesthesia (1.5–2% isoflurane inhalation) with an elastic string. This tagging method has been validated in previous AR-LABO studies and does not affect sociability [9]. For each behavioral assessment, four age- and sex-matched littermate C57BL/6J mice were placed together in a novel cage (276 × 445 × 204 mm; CL-0128, CLEA Japan Inc.) and allowed to interact freely for 15 h, comprising a 3-h light phase and a 12-h dark phase. Recording began 3 h before lights off, so the 3-h light phase and subsequent 12-h dark phase corresponded to the animals’ regular 12:12-h light/dark cycle; the cycle was not shifted during testing. The dark phase was the primary observation period because mice are nocturnal and exhibit most spontaneous activity during this phase. The preceding 3-h light interval served as an ancillary observation window immediately before lights off and was not intended as a duration-matched comparator. Light- and dark-phase data were analyzed separately, and the unequal recording durations were not compared directly. All four mice were monitored at 20 frames/s using an infrared camera with infrared illumination. Video data were analyzed offline using an in-house tracking system (O’Hara & Co., Ltd.) [9]. The center of each mouse’s ID tag was detected in each frame and recorded as XY coordinates within the cage. Time-series coordinate data were exported as comma-separated value files. Subsequent processing was performed using Python 3.10.8, in-house programs, and a previously described algorithm [8]. Missing coordinates between detected frames were linearly interpolated. Locomotor activity was quantified as the total distance moved (in meters) by each mouse’s ID tag during a specified period. Total distance traveled was calculated as the sum of frame-to-frame displacements, with each displacement defined as the Euclidean distance between the same mouse’s positions in consecutive frames. For clarity, we use the following terminology consistently: “locomotor activity” refers exclusively to the total distance traveled by an individual mouse; “inter-mouse distance” refers to the distance between the identification tags of two mice; and “light phase” and “dark phase” replace “day” and “night.” We avoid the unqualified term “distance” to prevent confusion between these measures. Throughout, we use “sociability” to denote the tendency to approach and maintain social contact and “social interaction” to denote the measured contact events and their temporal organization; other categories of social behavior (e.g., aggression, territorial, or maternal behavior) were not assessed.

**Fig 1:**
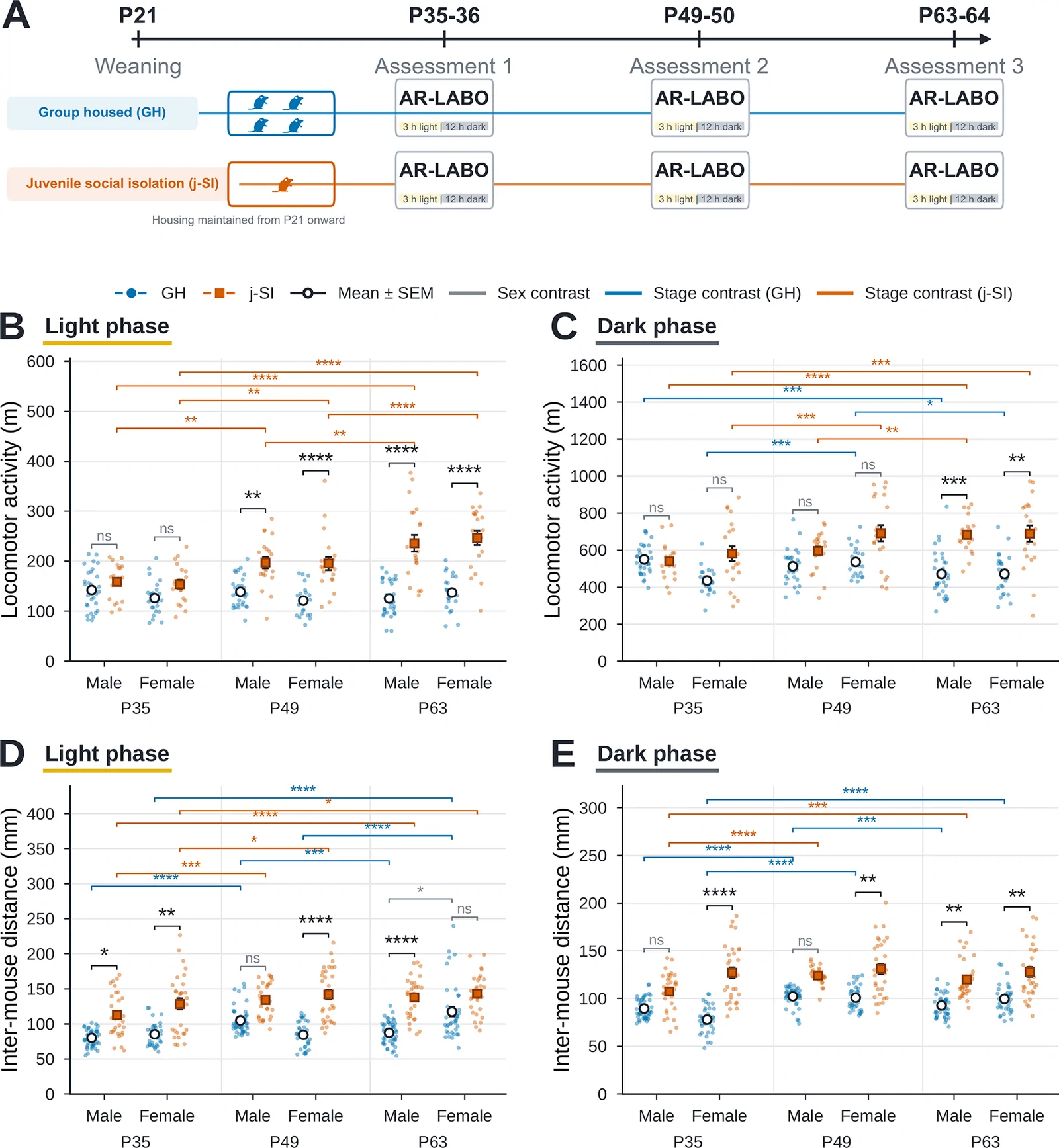
Experimental design and developmental, sex-, and housing-dependent changes in locomotor activity and inter-mouse distance. (A) Mice were weaned at postnatal day (P)21 and assigned to either group-housed (GH) or juvenile social isolation (j-SI) conditions throughout the experimental period. Behavioral assessments were performed with the automated tracking system (AR-LABO) in sessions beginning at P35, P49, and P63 and continuing into the following postnatal day. During each assessment period, spontaneous behavior was recorded for 3 h during the light phase and 12 h during the dark phase. (B) Light-phase locomotor activity (LMM stage × sex × housing interaction, Wald χ^2^(2) = 0.61, p = 0.739). j-SI mice traveled greater distances than GH mice at P49 (male: p = 0.001; female: p < 0.001) and P63 (both p < 0.001) but not at P35 (male: p = 1.000; female: p = 0.557). Within j-SI mice, distance traveled increased from P35 to P49 and from P49 to P63 in both sexes. (C) Dark-phase locomotor activity (χ^2^(2) = 9.04, p = 0.011). j-SI mice traveled greater distances than GH mice at P63 (male: p < 0.001; female: p = 0.001) but not at P35 or P49 (all p ≥ 0.054). Within GH mice, activity decreased from P35 to P63 in males, whereas in females it increased from P35 to P49 and decreased from P49 to P63; within j-SI mice, activity increased from P35 and P49 to P63 in males and from P35 to P49 and P63 in females. (D) Light-phase inter-mouse distance (χ^2^(2) = 27.47, p < 0.001). Distance was longer in j-SI than GH mice at P35 (male: p = 0.021; female: p = 0.002), at P49 in females (p < 0.001; male: p = 0.054), and at P63 in males (p < 0.001; female: p = 0.188). GH males had a shorter distance than GH females at P63 (p = 0.029). Distance increased from P35 to P49 and decreased from P49 to P63 in GH males, increased from P35 and P49 to P63 in GH females, and increased from P35 to P49 and P63 in j-SI males and females. (E) Dark-phase inter-mouse distance (χ^2^(2) = 26.73, p < 0.001). Distance was longer in j-SI than GH females at P35, P49, and P63 (p < 0.001, p = 0.004, and p = 0.009) and in j-SI males at P63 (p = 0.006; P35, p = 0.200; P49, p = 0.054). Distance increased from P35 to P49 and decreased from P49 to P63 in GH males, increased from P35 to P49 and P63 in GH females, and increased from P35 to P49 and P63 in j-SI males. Black brackets denote GH vs. j-SI contrasts, and gray brackets denote male vs. female contrasts within a housing condition; developmental-stage contrasts are described in the text of this legend. All comparisons are Bonferroni-adjusted contrasts of estimated marginal means from the primary LMM (see Statistical analysis; S2 Table; all contrasts, including stage contrasts, are provided in S1 Data). Statistical significance is indicated as *p < 0.05, **p < 0.01, ***p < 0.001, ****p < 0.0001; ns, not significant.

The AR-LABO analysis pipeline exported directed focal-mouse–partner records in which an initiated event for the focal mouse was recorded reciprocally as a received event for its partner. Social encounter count (Fig 2A, 2B) was defined as the number of approach-initiated events for each ordered focal–partner dyad using the 20-mm ID-tag threshold. Mean social contact duration per initiated encounter (Fig 2C, 2D) was calculated for each ordered dyad as the total approach-associated duration at the 30-mm threshold divided by the corresponding approach count; duration is reported in seconds. Inter-mouse distance is measured between dorsal ID tags rather than body boundaries, so these thresholds indicate close spatial proximity but not body-boundary distance. Entropy and switching-ratio analyses were based on 20-mm initiated-contact records. Values that are undefined by construction were treated as missing in all analyses: switching ratio when a mouse had fewer than two initiated contacts in a phase (coded −1 by the source pipeline; n = 18 light-phase mouse-stage observations), interaction-partner entropy when a mouse had no initiated contact (n = 9 light-phase observations), and mean contact duration per initiated encounter when a directed dyad had no initiated encounter (n = 12 light-phase dyad-stage observations). All of these observations were from j-SI mice. In the previously submitted analyses, these values had been entered as −1 or 0; all affected models, contrasts, and figures were recalculated.

**Fig 2:**
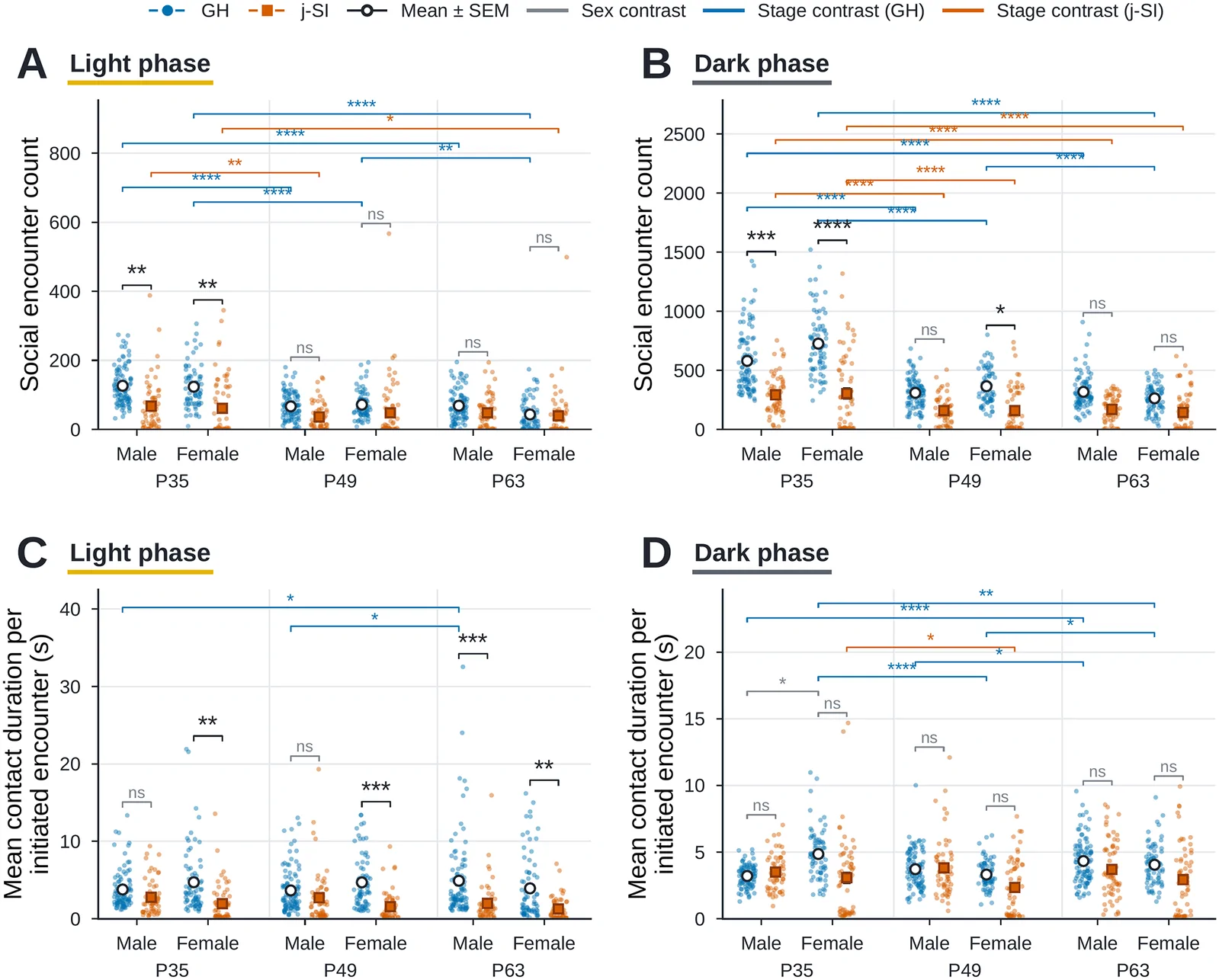
Social encounter frequency and mean contact duration per initiated encounter across development, sex, and light/dark phase. (A) Light-phase social encounter count (LMM stage × sex × housing interaction, Wald χ^2^(2) = 1.43, p = 0.488). j-SI mice had fewer encounters than GH mice at P35 (male: p = 0.008; female: p = 0.009) but not at P49 or P63 (all p ≥ 0.602). Encounters decreased from P35 to P49 and P63 in GH males and females and further from P49 to P63 in GH females; in j-SI mice, they decreased from P35 to P49 in males and from P35 to P63 in females. (B) Dark-phase social encounter count (χ^2^(2) = 10.51, p = 0.005). j-SI mice had fewer encounters than GH mice at P35 in both sexes (both p < 0.001) and at P49 in females (p = 0.031); differences at P49 and P63 in males and at P63 in females were not significant (p = 0.185, 0.196, and 0.623). Encounters decreased from P35 to P49 and P63 in all housing × sex groups and further from P49 to P63 in GH females. (C) Light-phase mean contact duration per initiated encounter (χ^2^(2) = 6.30, p = 0.043). j-SI females had shorter durations than GH females at P35 (p = 0.006), P49 (p < 0.001), and P63 (p = 0.009); j-SI males had shorter durations than GH males at P63 (p < 0.001) but not at P35 or P49 (both p = 1.000). Duration increased from P35 and P49 to P63 in GH males. Directed dyads without an initiated encounter (n = 12, all j-SI) have no defined duration and were excluded. (D) Dark-phase mean contact duration per initiated encounter (χ^2^(2) = 9.67, p = 0.008). No GH vs. j-SI contrast was significant (female P35: p = 0.050; all others p ≥ 0.581). GH males had a shorter duration than GH females at P35 (p = 0.038). Duration increased from P35 and P49 to P63 in GH males; in GH females it decreased from P35 to P49 and P63 and increased from P49 to P63; in j-SI females it decreased from P35 to P49. Encounter counts (A,B) use 20-mm initiated events; durations (C,D) use 30-mm approach-associated duration divided by approach count and are shown in seconds. Black brackets denote GH vs. j-SI contrasts, and gray brackets denote male vs. female contrasts within a housing condition; developmental-stage contrasts are described in the text of this legend. All comparisons are Bonferroni-adjusted contrasts of estimated marginal means from the primary LMM (see Statistical analysis; S2 Table; all contrasts, including stage contrasts, are provided in S1 Data). Statistical significance is indicated as *p < 0.05, **p < 0.01, ***p < 0.001, ****p < 0.0001; ns, not significant.

To assess whether a mouse concentrated contacts on a specific cage mate, an entropy measure (*H*_*i*_**)** was calculated from the probability (*p*_*ij*_) of contacting different mice using the following formula:


$$\begin{array}{c} H_{i}=-\Sigma_{j}\left\{p_{ij}{log}_{2}\left(p_{ij}\right)\right\}, \end{array}$$ (1)

where Hi denotes the entropy of social interactions for mouse i; pij is the probability of mouse i interacting with mouse j (either in terms of number of interactions or total interaction duration); and the summation ∑j runs over all potential interaction partners j (i.e., the three available interaction partners of mouse i). This dimensionless Shannon entropy measure quantified how evenly contacts were distributed across interaction partners.

We calculated a switching ratio from the temporal sequence of interaction-partner identities to quantify how often mice changed social partners over time. Each mouse’s time-ordered contact history was represented as a vector of interaction-partner IDs of length *n*. Consecutive repetitions of the same partner were collapsed into a reduced sequence—for example, the sequence (3,3,3,3,3,3,3,3) was reduced to (3), whereas (3,3,3,3,4,4,4,4) was reduced to (3,4). The switching ratio was then defined as:


$$\begin{array}{c} \text{switching ratio}=\frac{\left(\text{m}-\text{1}\right)}{\left(n-\text{1}\right)}, \end{array}$$ (2)

where m denotes the number of elements in the reduced sequence and n denotes the length of the original sequence. This normalization constrains the switching ratio between 0 and 1—a value of 0 indicates no switching (continuous interaction with the same partner); a value of 1 indicates maximal switching, in which the interaction partner changes at every possible transition; and intermediate values represent partial switching behavior. This metric therefore captures organizational differences in social interactions that are not reflected by total interaction time alone.

Exploratory directional analyses used available light-phase files from the analysis archive. For each mouse and stage, A and R denoted its 20-mm approach and receive counts. A signed initiation index, (A − R)/(A + R), tested whether a mouse tended to initiate rather than receive events overall, whereas its absolute value quantified the magnitude of individual directional specialization independent of sign. Analogous indices were calculated from the total 30-mm approach and receive durations. Because each initiated event for one mouse is necessarily a received event for another mouse, approach and receive totals are identical at the four-mouse-group level; analyses therefore focused on individual imbalance indices.

Partner specificity was assessed using the light-phase 20-mm directed approach-count matrix. For each mouse, the maximum partner share was the largest fraction of initiated events directed to any one of its three partners, and the Herfindahl index was the sum of squared partner shares (1/3 for an even distribution and 1 for complete concentration on one partner). The preferred partner was the partner with the largest count; ties were treated as missing for persistence analyses. Preferred-partner identity was compared across P35–P49, P49–P63, and all three stages. These measures characterize partner concentration and identity but cannot resolve dominance or partner traits, which were not recorded.

Within-session switching dynamics were examined using the time-binned files in the archive: six consecutive 30-min bins during the 3-h light phase and twelve consecutive 60-min bins during the 12-h dark phase. Values coded −1 by the source pipeline indicated undefined switching because too few valid interactions occurred; these values were treated as missing. For each mouse-session with at least three valid bins, the slope of switching ratio against elapsed time (hours) was estimated. The first light-phase 30-min bin was also contrasted with the mean of the later valid light-phase bins. These exploratory data do not resolve the first 5–10 min and provide no time courses for the other outcomes.

### Statistical analysis

All statistical analyses were performed separately for the light and dark phases. The primary longitudinal analyses used Gaussian linear mixed-effects models (LMMs) with developmental stage, sex, and housing condition and all two- and three-way interactions as fixed effects. Random intercepts were specified for the four-mouse test group (cage) and the analysis unit nested within cage: individual mouse for locomotor activity, entropy, and switching; undirected dyad for inter-mouse distance; and directed focal–partner dyad for encounter count and contact duration. The dataset comprised 24 independent four-mouse groups (8 male GH, 5 male j-SI, 6 female GH, and 5 female j-SI), 96 mice, 144 undirected dyads, and 288 directed dyads, each measured at P35, P49, and P63. Variance components and fixed effects were estimated by restricted maximum likelihood (REML); joint fixed-effect terms were evaluated using Wald χ^2^ tests. Outcomes were standardized within each measure and phase before fitting; this standardization does not alter non-intercept fixed-effect tests. The cage random intercept was tested by refitting models by maximum likelihood (ML), comparing the full model with a model omitting cage, and using the 0.5χ^2^0 + 0.5χ^2^1 boundary reference for the likelihood-ratio p value. The same random-effects structure and Wald tests were used for exploratory directional, partner-specificity, and time-course outcomes. Fisher’s exact tests descriptively compared preferred-partner persistence proportions. Planned contrasts of LMM estimated marginal means were tested with Wald z tests: GH vs. j-SI at each developmental stage within each sex (Bonferroni adjustment for six comparisons per outcome), male vs. female within each housing condition and stage (six comparisons per outcome), and pairwise developmental-stage comparisons within each housing × sex group (three comparisons per group). Housing and sex contrasts are annotated in Figs 1–3 and reported in S2 Table; developmental-stage contrasts are described in the figure legends; all contrasts are provided in S1 Data. Data are presented as mean ± SEM, and significance was set at p < 0.05 (two-tailed). Complete model results are reported in S1–S3 Tables and S1 Data, and cage and analysis-unit identifiers are provided in S1 Data.

**Fig 3:**
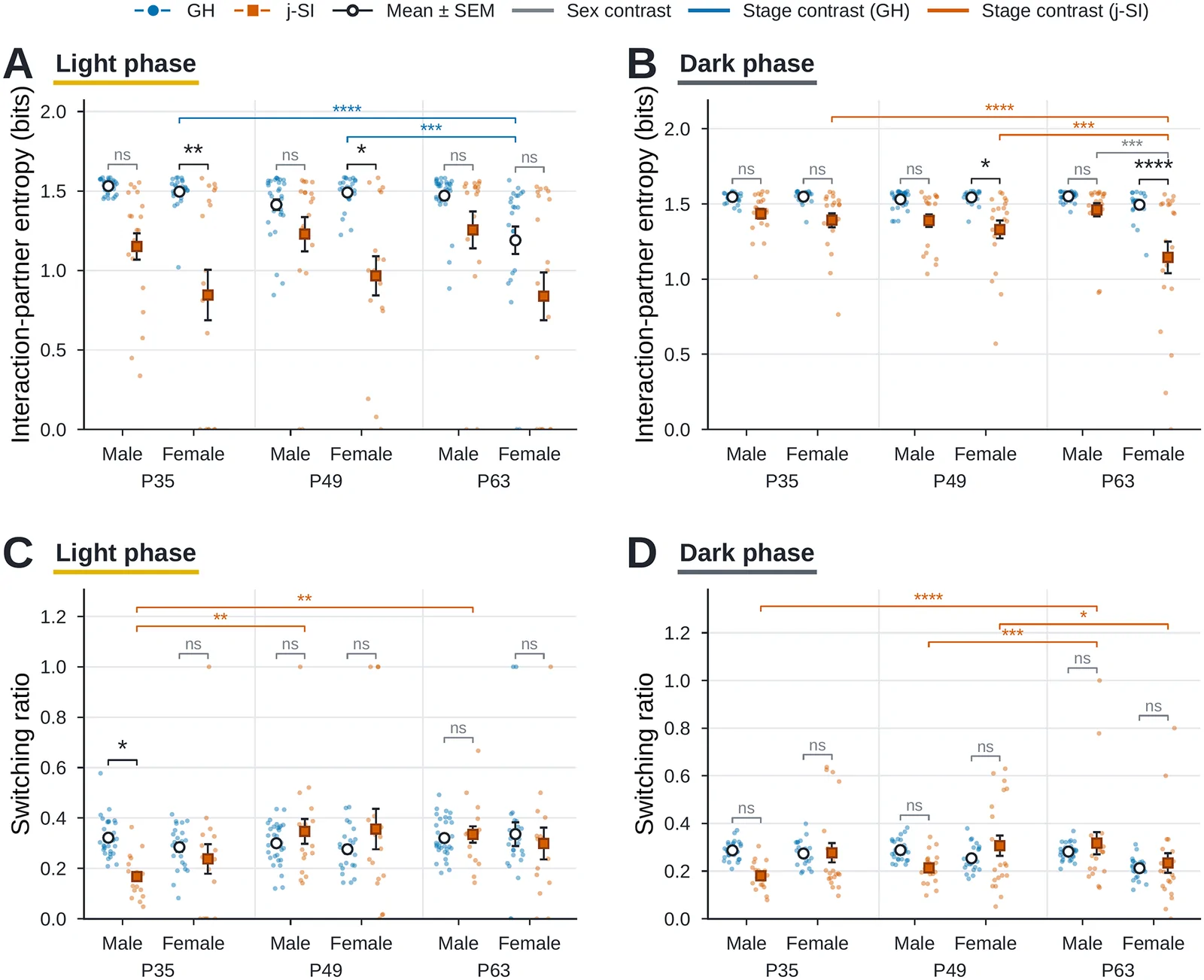
Social interaction structure assessed by interaction-partner entropy and switching ratio. (A) Light-phase interaction-partner entropy (LMM stage × sex × housing interaction, Wald χ^2^(2) = 4.13, p = 0.127; housing, χ^2^(1) = 10.70, p = 0.001). j-SI females had lower entropy than GH females at P35 (p = 0.003) and P49 (p = 0.015) but not at P63 (p = 0.360); no housing contrast was significant in males (all p ≥ 0.266). In GH females, entropy decreased from P35 and P49 to P63. Mouse-stage observations without an initiated contact (n = 9, all j-SI) have undefined entropy and were excluded. (B) Dark-phase entropy (χ^2^(2) = 6.53, p = 0.038). j-SI females had lower entropy than GH females at P49 (p = 0.017) and P63 (p < 0.001); no housing contrast was significant in males (all p ≥ 0.208). At P63, j-SI females had lower entropy than j-SI males (p < 0.001), and entropy in j-SI females decreased from P35 and P49 to P63. (C) Light-phase switching ratio (χ^2^(2) = 2.81, p = 0.245; stage × housing, χ^2^(2) = 11.66, p = 0.003). j-SI males had a lower switching ratio than GH males at P35 (p = 0.011); no other housing contrast was significant (all p ≥ 0.782). In j-SI males, switching increased from P35 to P49 and P63. Observations with undefined switching (source value −1, fewer than two initiated contacts; n = 18, all j-SI) were excluded. (D) Dark-phase switching ratio (χ^2^(2) = 8.63, p = 0.013). No GH vs. j-SI contrast was significant (male P35: p = 0.115; all others p ≥ 0.595). In j-SI males, switching increased from P35 and P49 to P63; in j-SI females, it decreased from P49 to P63. Black brackets denote GH vs. j-SI contrasts, and gray brackets denote male vs. female contrasts within a housing condition; developmental-stage contrasts are described in the text of this legend. All comparisons are Bonferroni-adjusted contrasts of estimated marginal means from the primary LMM (see Statistical analysis; S2 Table; all contrasts, including stage contrasts, are provided in S1 Data). Statistical significance is indicated as *p < 0.05, **p < 0.01, ***p < 0.001, ****p < 0.0001; ns, not significant.

To characterize within-subject developmental trajectories and maintain continuity with the broader behavioral literature, the same outcomes were also analyzed with three-way repeated-measures analysis of variance (RM-ANOVA), with developmental stage (P35, P49, P63) as a within-subject factor and sex and housing condition as between-subject factors, using analysis units with complete data at all three stages. When sphericity was violated, the Greenhouse–Geisser correction was applied. Thus, RM-ANOVA addressed the complementary question of within-animal change across development, whereas the LMM provided the primary inference, including all post hoc contrasts, while accounting for the clustering structure. Omnibus RM-ANOVA results are reported in S1 Table; LMM results are reported in the Results and S1 Table.

## Results

### Developmental, sex-dependent, and housing-dependent effects on locomotor activity and inter-mouse distance

In the primary LMM, the developmental stage × sex × housing interaction was not significant for light-phase locomotor activity (Wald χ^2^(2) = 0.61, p = 0.739) but was significant during the dark phase (χ^2^(2) = 9.04, p = 0.0109). The interaction was significant for inter-mouse distance during both the light phase (χ^2^(2) = 27.47, p < 0.001) and dark phase (χ^2^(2) = 26.73, p < 0.001) (S1 Table). LMM contrasts are reported below, and complementary RM-ANOVA results are summarized in S1 Table. Inter-mouse distance is presented with locomotor activity because both measures derive from continuous positional data and jointly describe how mice distributed themselves within the cage. Inter-mouse distance is a spatial-proximity index influenced by locomotion, whereas encounter count and mean contact duration per initiated encounter—analyzed below—are direct summaries of interaction events.

LMM contrasts showed that light-phase locomotor activity was greater in j-SI than in GH mice at P49 and P63 in both sexes (males: + 58.2 m, p = 0.001, and +110.6 m, p < 0.001; females: + 74.0 m and +109.3 m, both p < 0.001), with no housing difference at P35 (Fig 1B). During the dark phase, j-SI mice showed greater locomotor activity than GH mice only at P63 (males: + 211.9 m, p < 0.001; females: + 218.2 m, p = 0.001) (Fig 1C). Within j-SI mice, locomotor activity increased from P35 to P63 in both sexes and phases, whereas within GH mice dark-phase activity decreased from P35 to P63 in males and increased from P35 to P49 in females.

Light-phase inter-mouse distance was longer in j-SI than in GH mice in males at P35 (+32.7 mm, p = 0.021) and P63 (+50.2 mm, p < 0.001; P49, p = 0.054) and in females at P35 (+43.0 mm, p = 0.002) and P49 (+57.4 mm, p < 0.001), but not at P63 (p = 0.188) (Fig 1D). During the dark phase, inter-mouse distance was longer in j-SI females at all three stages (+49.0, + 30.3, and +28.4 mm; p < 0.001, p = 0.004, and p = 0.009) and in j-SI males at P63 only (+27.4 mm, p = 0.006) (Fig 1E). Among GH mice at P63, light-phase inter-mouse distance was shorter in males than in females (−29.8 mm, p = 0.029); no other sex contrast was significant for locomotor activity or inter-mouse distance. Complementary RM-ANOVA showed significant housing main effects for all four outcomes and significant stage × sex × housing interactions for dark-phase locomotor activity and for inter-mouse distance in both phases (S1 Table).

Together, locomotor activity and inter-mouse distance showed distinct associations with developmental stage, sex, and housing condition. Light-phase locomotor activity was influenced primarily by housing condition, whereas dark-phase activity showed a significant three-way interaction among developmental stage, sex, and housing condition. In contrast, inter-mouse distance showed consistent housing-dependent differences across both phases, whose stage-specific expression differed by sex and phase.

### Developmental, sex-dependent, and phase-dependent modulation of social encounter frequency and mean contact duration per initiated encounter

In the primary LMM, the developmental stage × sex × housing interaction was not significant for light-phase social encounter count (Wald χ^2^(2) = 1.43, p = 0.488) but was significant during the dark phase (χ^2^(2) = 10.51, p = 0.0052). The interaction was significant for mean contact duration per initiated encounter during both the light phase (χ^2^(2) = 6.30, p = 0.0429) and dark phase (χ^2^(2) = 9.67, p = 0.0080) (S1 Table). LMM contrasts are reported below, and complementary RM-ANOVA results are summarized in S1 Table.

LMM contrasts showed that j-SI mice had fewer light-phase social encounters than GH mice at P35 in both sexes (males: −59.1, p = 0.008; females: −62.3, p = 0.009), with no housing difference at P49 or P63 (all p ≥ 0.602) (Fig 2A). During the dark phase, j-SI mice had fewer encounters than GH mice at P35 in both sexes (males: −287.8; females: −421.7; both p < 0.001) and at P49 in females (−205.1, p = 0.031); the housing differences at P49 and P63 in males and at P63 in females were not significant (p = 0.185, 0.196, and 0.623, respectively) (Fig 2B). Encounter counts decreased from P35 to later stages in most housing × sex groups in both phases. Light-phase mean contact duration per initiated encounter was shorter in j-SI than in GH females at all three stages (−2.77, −3.21, and −2.70 s; p = 0.006, p < 0.001, and p = 0.009) and in j-SI males at P63 (−3.01 s, p < 0.001) (Fig 2C). No housing contrast was significant for dark-phase mean contact duration (females at P35: −1.79 s, p = 0.050; all other p ≥ 0.581); among GH mice at P35, dark-phase mean contact duration was shorter in males than in females (−1.65 s, p = 0.038) (Fig 2D). Complementary RM-ANOVA showed significant housing main effects for all four outcomes and stage × sex × housing interactions for dark-phase encounter count and dark-phase mean contact duration (S1 Table).

Together, social encounter frequency and mean contact duration per initiated encounter showed distinct associations with developmental stage, sex, and housing condition across the light and dark phases. Light-phase encounter counts showed developmental-stage and housing effects without a three-way interaction, whereas the dark phase showed a significant three-way interaction across developmental stage, sex, and housing condition. Mean contact duration per initiated encounter was consistently shorter in j-SI females during the light phase, whereas no dark-phase housing contrast was significant.

### Developmental, sex-dependent, and phase-dependent modulation of social interaction structure

In the primary LMM, the developmental stage × sex × housing interaction was not significant for interaction-partner entropy during the light phase (Wald χ^2^(2) = 4.13, p = 0.127), for which the housing effect (χ^2^1) = 10.70, p = 0.001) and the stage × sex (χ^2^(2) = 7.41, p = 0.025) and stage × housing (χ^2^(2) = 7.25, p = 0.027) interactions were significant, but it was significant during the dark phase (χ^2^(2) = 6.53, p = 0.0382). The interaction was not significant for switching ratio during the light phase (χ^2^(2) = 2.81, p = 0.245), for which the stage × housing interaction was significant (χ^2^(2) = 11.66, p = 0.003) and the housing effect was not (χ^2^(1) = 0.22, p = 0.639), but it was significant during the dark phase (χ^2^(2) = 8.63, p = 0.0134) (S1 Table). LMM contrasts are reported below, and complementary RM-ANOVA results are summarized in S1 Table.

LMM contrasts showed lower light-phase interaction-partner entropy in j-SI than in GH females at P35 (−0.70 bits, p = 0.003) and P49 (−0.61 bits, p = 0.015), but not at P63 (p = 0.360); no housing contrast was significant in males (all p ≥ 0.266) (Fig 3A). During the dark phase, entropy was lower in j-SI than in GH females at P49 (−0.21 bits, p = 0.017) and P63 (−0.35 bits, p < 0.001), and at P63 j-SI females had lower entropy than j-SI males (by 0.32 bits, p < 0.001); no housing contrast was significant in males (all p ≥ 0.208) (Fig 3B). The light-phase switching ratio was lower in j-SI than in GH males at P35 (−0.154, p = 0.011) and then increased within j-SI males from P35 to P49 (+0.184, p = 0.001) and P63 (+0.173, p = 0.004); no other housing contrast was significant (all p ≥ 0.782) (Fig 3C). For the dark-phase switching ratio, no housing contrast reached significance (males at P35: −0.106, p = 0.115; all other p ≥ 0.595), and switching increased within j-SI males from P35 and P49 to P63 (both p < 0.001) (Fig 3D). Complementary RM-ANOVA showed stage × sex × housing interactions for light-phase entropy and dark-phase switching ratio, but not for dark-phase entropy or light-phase switching ratio (S1 Table).

Together, social interaction structure, as assessed by interaction-partner entropy and switching ratio, showed phase-dependent associations with developmental stage, sex, and housing condition. Lower interaction-partner entropy after j-SI was detected in females (light phase at P35 and P49; dark phase at P49 and P63) but not in males, whereas lower switching was confined to males at P35 during the light phase. In the primary LMM, the three-way interaction was significant for both measures during the dark phase but not during the light phase, although the phases were analyzed separately.

The cage random intercept was supported in 11 of 12 outcome-by-phase models (ML likelihood-ratio χ^2^(1) = 10.76–129.02, all boundary-adjusted p < 0.001; S1 Table), indicating residual between-cage variation after accounting for fixed effects and repeated analysis units; the exception was light-phase switching ratio (χ^2^(1) = 0.57, p = 0.226). The housing fixed effect was significant in 9 of 12 models; the exceptions were dark-phase contact duration (χ^2^(1) = 2.44, p = 0.118), light-phase switching ratio (χ^2^(1) = 0.22, p = 0.639), and dark-phase switching ratio (χ^2^(1) = 0.15, p = 0.700).

### Exploratory directionality, partner specificity, and within-session switching

In light-phase directional records, the signed initiation index did not differ from zero overall (Wald χ^2^(1) = 1.36, p = 0.243) and showed no housing effect (χ^2^(1) = 1.41, p = 0.235), indicating no overall shift toward initiating rather than receiving events. However, the absolute count imbalance was larger in j-SI mice than in GH mice (descriptive means, 0.365 vs 0.194; housing χ^2^(1) = 12.56, p < 0.001), indicating greater individual specialization toward relatively more initiating or receiving roles. The corresponding signed duration index showed no housing effect (p = 0.308); the absolute duration-imbalance housing effect was not significant (p = 0.093), although its stage × housing interaction was significant (p < 0.001) (S3 Table).

Partner-specific light-phase records showed a greater concentration of initiated events in j-SI mice. The maximum share directed to one partner averaged 0.637 in j-SI mice and 0.484 in GH mice (housing χ^2^(1) = 13.27, p < 0.001), and the Herfindahl index averaged 0.557 and 0.396, respectively (housing χ^2^(1) = 11.15, p < 0.001). Stage × housing interactions were also significant for maximum share (p = 0.024) and Herfindahl index (p = 0.042). Preferred-partner identity showed no greater persistence across stages in j-SI mice: all sex-specific Fisher’s exact comparisons for P35–P49, P49–P63, and all three stages were non-significant (all p ≥ 0.191). Partner dominance and other partner traits were not recorded (S3 Table).

Switching ratios declined within sessions. The marginal light-phase slope was −0.070 per hour (SE = 0.007, Wald χ^2^(1) = 96.21, p < 0.001), and the dark-phase slope was −0.0102 per hour (SE = 0.0020, χ^2^(1) = 24.71, p < 0.001). During the light phase, switching in the first 30 min exceeded the mean of the later valid bins by 0.139 (SE = 0.013, χ^2^(1) = 108.94, p < 0.001); this contrast showed a housing effect (p = 0.0125) and a stage × sex × housing interaction (p = 0.0299). Because undefined bins were more frequent when interactions were sparse, particularly in j-SI records, these bin-level time-course findings are exploratory (Fig 4; S3 Table).

**Fig 4:**
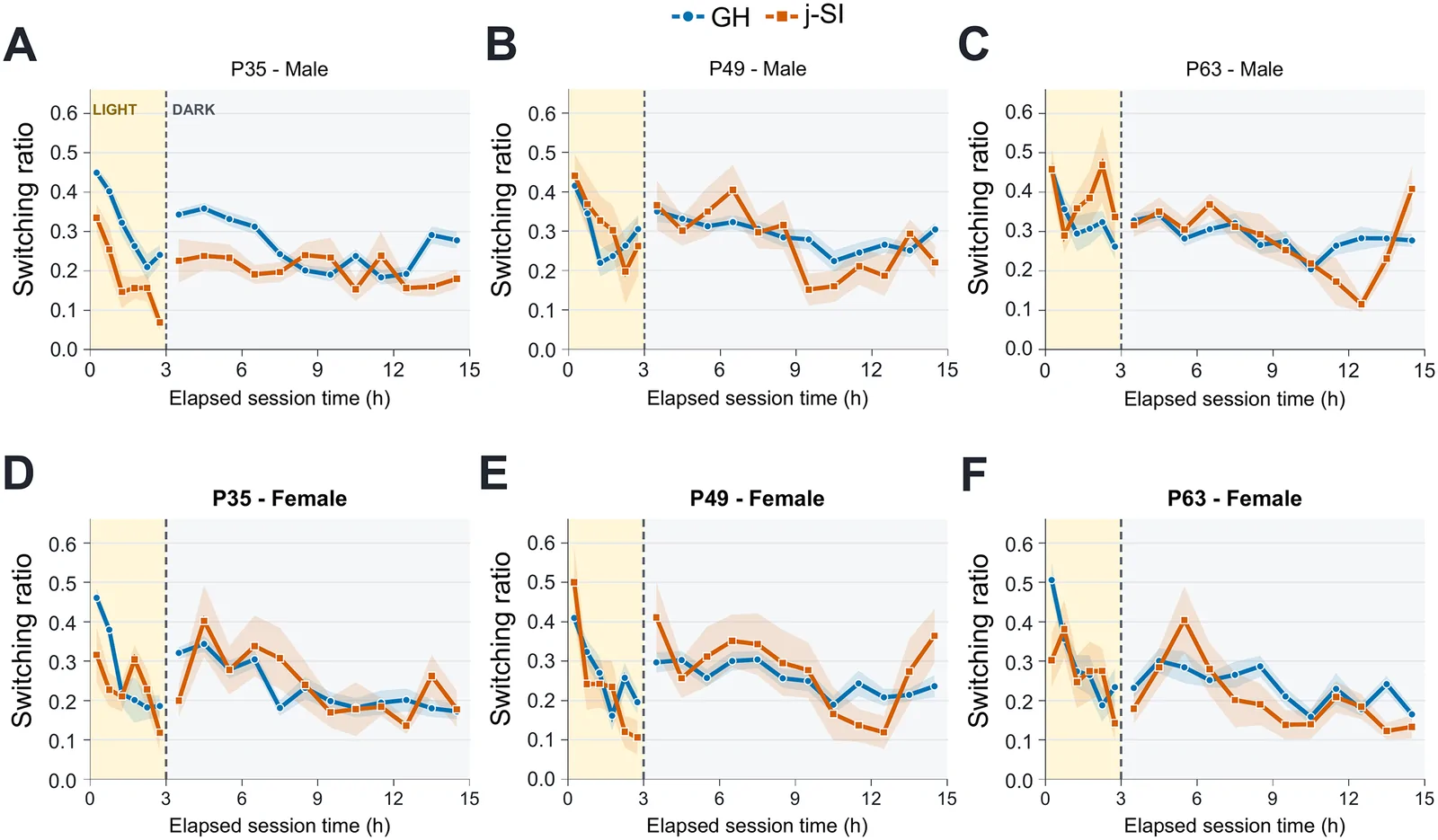
Switching ratio across six 30-min light-phase bins and twelve 60-min dark-phase bins. Lines and shading indicate mean ± SEM for valid observations; source values of −1 (undefined switching due to insufficient interactions) were treated as missing. The vertical dashed line marks lights off and the start of the dark phase.

## Discussion

In this study, continued post-weaning social isolation was associated with persistent but developmentally dynamic and sex-specific alterations in social behavior. Rather than producing a simple increase or decrease in overall sociability, j-SI was associated with multidimensional changes in social spacing, contact persistence, and temporal organization of social interactions, as captured by prolonged cage-based monitoring across developmental stages. Across the three developmental stages, j-SI mice generally showed greater inter-mouse distances than GH mice, although not in every sex–phase combination; mean contact duration per initiated encounter varied more clearly by sex, stage, and phase. Differences were observed in both the light and dark phases, but the phases were analyzed separately and should not be interpreted as directly comparable effects.

Most previous research has relied on short-duration dyadic tests that primarily capture initial social approach and do not reflect the spatial and temporal organization of group-based social behavior. We therefore used prolonged automated tracking to continuously monitor multiple animals, enabling analysis of social spacing, contact dynamics, and interaction patterns across development.

We used prolonged post-weaning isolation that extended beyond the classical P21–P35 j-SI window. Isolation began at P21, consistent with the j-SI paradigm, but individual housing continued beyond P35. The later behavioral outcomes therefore reflect the combined effects of early-life isolation and continued post-weaning social deprivation. Here, j-SI refers to isolation beginning during the juvenile critical period and continuing through subsequent development.

### Sex-specific organization of social interaction after j-SI

Despite these shared features, males and females differed in the temporal organization of social behavior. In male mice, j-SI was associated with a lower light-phase switching ratio and fewer social encounters in both phases at P35, differences that were no longer evident at P63; by P63, housing differences in males were expressed mainly as greater locomotor activity, greater inter-mouse distance, and shorter light-phase mean contact duration per initiated encounter (Figs 1–3). These findings complement prior reports that post-weaning or adolescent isolation can increase aggression, alter social preference, and disrupt prefrontal network organization in males [20–22]. However, partner switching itself was not directly assessed in those studies and should therefore be regarded as a novel descriptive feature of the present dataset.

In contrast, j-SI females showed greater inter-mouse distance at most stages in both phases, shorter light-phase mean contact duration per initiated encounter at all three stages, and lower interaction-partner entropy (light phase at P35 and P49; dark phase at P49 and P63), without housing differences in switching ratio (Figs 1–3). This pattern is consistent with evidence that post-weaning isolation decreases social preference in young-adult females [22], while other studies have demonstrated sex-dependent neural and physiological responses to isolation [18,25,27,31–33]. Together, these findings suggest that responses to early social deprivation involve distinct, sex-specific patterns of social behavior rather than a single behavioral dimension.

### Reinterpreting sociability after juvenile isolation

Previous studies using discrete social preference paradigms have reported increased sociability or hypersocial behavior after post-weaning or adolescent isolation in males and, in one study, both sexes [21,22], whereas resident–intruder studies have reported increased aggression in isolated males [20]. However, these paradigms assess brief interactions with a single social stimulus or intruder and therefore capture only limited dimensions of social behavior [23,24,34,35]. In contrast, we assessed spontaneous social behavior in a group over extended periods, enabling evaluation of social spacing, contact persistence, and the temporal organization of interactions. Under these conditions, our data indicate that, over the full recording period, isolated males did not show enhanced overall sociability; instead, juvenile isolation was associated with greater inter-mouse distance, stage- and sex-dependent reductions in mean contact duration per initiated encounter, and altered interaction dynamics. The primary outcomes summarize behavior at the phase level, but the exploratory switching time course showed higher switching in the first 30 min followed by a within-session decline. This pattern indicates an early-to-later change in interaction sequencing; however, the available 30-min bins cannot resolve the first 5–10 min, no comparable time courses were available for approach count, contact duration, or locomotion, and aggression was not directly measured. Our positional tracking likewise cannot distinguish reduced sociability from altered responses to less familiar conspecifics, such as increased vigilance, territorial responding, or aggression, which social isolation is known to potentiate in males. We therefore limit our conclusion to overall sociability under the conditions tested and explicitly acknowledge acute social re-exposure and aggression/territoriality as alternative or additional contributors that the present design cannot rule out. In males, altered switching behavior and activity patterns may reflect greater social reactivity or instability in interaction sequencing rather than elevated social motivation per se; because prior studies did not quantify partner switching, this interpretation remains tentative. These findings are consistent with evidence that post-weaning social isolation alters prefrontal and reward-related circuitry [7–9,16,20,25,36] and with reports of sex-dependent neural responses to isolation [18,25,27,32,33].

This interpretation is consistent with previous reports showing that the behavioral consequences of social isolation vary with sex, developmental timing, and behavioral assay [19,21–24,34,37]. Brief preference-based assays primarily index initial social approach toward a single stimulus [23,24,34]. By contrast, prolonged group-based assessments capture the spatial and temporal organization of social behavior [28,29], including inter-mouse distance, mean contact duration per initiated encounter, and partner switching, which reflect sustained and dynamically regulated interactions not readily captured by brief tests.

The entropy and switching measures used here require careful interpretation. Both are model-free descriptors of social-contact organization: entropy quantifies how evenly an animal distributes contacts across its three partners, whereas switching quantifies how frequently it alternates partners over time. Neither dimension is represented by total contact time or encounter frequency alone, and the two indices were dissociable. Direct partner-level analysis confirmed that j-SI mice concentrated a larger share of initiated light-phase events on a single partner, supporting a less even distribution of contacts among partners. However, preferred-partner identity was not consistently more stable across development, and the dataset contains no dominance rank, genotype, hormonal state, or other partner trait with which to characterize the selected individual. Entropy, switching, and partner-concentration measures were not independently validated behaviorally in this study and should therefore remain descriptive, hypothesis-generating indices.

The divergence between light- and dark-phase findings also requires careful interpretation. Mice are nocturnal, and the dark phase corresponds to their primary period of spontaneous activity; dark-phase measures therefore reflect behavior during a high-activity period. The stage × sex × housing interactions detected during the dark phase, but not the light phase, for locomotor activity, encounter counts, and switching ratio suggest that isolation-related differences are most evident when animals are most active, whereas the shorter light interval indexes behavior during a lower-activity, late-light-phase window. Because the two phases were analyzed separately and are not directly comparable, the phase-dependent pattern suggests that behavioral differences associated with juvenile isolation are most evident during periods of high spontaneous activity. Greater inter-mouse distance is interpreted here as reduced spatial proximity, but proximity is ambiguous in valence: reduced proximity may reflect diminished affiliation or, alternatively, active avoidance or territorial spacing toward a less familiar conspecific, and our positional data cannot distinguish these.

### Developmental timing under continued isolation

The developmental timing of these findings is notable. Altered social spacing was evident at P35, locomotor differences became more pronounced at P49 and P63, and lower switching in males was confined to P35. The later-emerging locomotor differences are compatible with the possibility that juvenile stress has latent effects that continue to manifest as social circuits mature during adolescence and early adulthood [38–42]. This interpretation is consistent with human and animal evidence that adverse social experiences during sensitive periods can bias subsequent developmental trajectories [12,13,40–44]. Our longitudinal design therefore illustrates the value of assessing behavior at multiple developmental stages because a single time point could obscure sex-specific vulnerabilities or adaptive responses. However, later behavioral changes may also reflect the cumulative effects of prolonged isolation rather than solely latent consequences of early-life isolation. Because isolation continued beyond the initial post-weaning period, the observed phenotypes may reflect both early-life disruption during a sensitive period and continued social deprivation across development. In addition, the longitudinal design included three prolonged periods of intensive social interaction (at P35, P49, and P63), during which otherwise isolated mice experienced 15 h of group housing. These repeated re-exposures could influence the developmental trajectory of social behavior—for example, by providing intermittent social experience or by cumulative habituation to the test context—so the later time points reflect both continued isolation and prior testing sessions.

### Limitations and implications

This study has several limitations. First, AR-LABO quantifies spontaneous spatial and interaction patterns, but dorsal-marker tracking cannot determine social motivation or directly score biting, tail rattling, chasing, wrestling, dominance, or aggression; increased activity and spacing are compatible with, but not diagnostic of, vigilance or aggression. Second, hormonal status and the estrous cycle were not controlled. Third, GH mice interacted with continuously familiar cage mates, whereas j-SI mice were reunited with littermates after more than two weeks of separation and were effectively unfamiliar; the effects of reduced familiarity are therefore inseparable from those of isolation, and a familiarity-matched control would require testing both groups with unfamiliar conspecifics. Fourth, only switching ratio was available at subphase resolution, in six 30-min light bins and twelve 60-min dark bins. These data demonstrate an early-to-later decline in switching but cannot resolve the first 5–10 min or time courses for other measures, and undefined switching bins were more common when contacts were sparse. Fifth, partner identity and directionality could be analyzed from the light-phase processed files, but no partner characteristics or dominance ranks were recorded, and corresponding dark-phase directional/partner matrices were unavailable. Sixth, observations within each four-mouse group were not independent. The LMMs incorporated cage and nested analysis-unit random intercepts, but the study included only 24 independent groups, limiting cluster-level precision and generalizability. In addition, switching ratio, entropy, and mean contact duration per initiated encounter are undefined when too few initiated contacts occur; these observations, all from j-SI mice, were treated as missing, so estimates for the sparsest-contact animals rest on fewer observations. Seventh, isolation continued through the final assessment, so the design cannot separate time-limited P21–P35 isolation from continued deprivation. Repeated 15-h social test sessions could also influence later behavior. A resocialization group, familiarity-matched controls, video ethology, and higher-resolution event data would help to separate these components. Accordingly, “persistent” denotes differences observed under ongoing isolation, not irreversibility after resocialization.

## Conclusions

Our results indicate that continued j-SI is associated with persistent, sex-specific changes in sociability and social interaction across development. Specifically, j-SI was associated with greater inter-mouse distance, shorter mean contact duration per initiated encounter under some conditions, and altered interaction dynamics, with males and females showing distinct developmental trajectories. These findings highlight adolescence as a sensitive period for social experience-dependent behavioral development and emphasize the need to consider sex, developmental timing, and behavioral context when evaluating the long-term behavioral consequences of post-weaning social deprivation.

## Supporting information

S1 Data Processed primary and exploratory data, cage and analysis-unit identifiers, complete LMM outputs, all LMM contrasts (including developmental-stage contrasts), and RM-ANOVA outputs for Figs 1–4. (XLSX)

S1 Table Primary linear mixed-effects model results (random effects, cage likelihood-ratio tests, and fixed-effect Wald tests) and complementary RM-ANOVA omnibus results for all behavioral outcomes. (DOCX)

S2 Table Bonferroni-adjusted LMM contrasts between housing conditions and between sexes (Figs 1–3). (DOCX)

S3 Table Exploratory light-phase directionality, partner-specificity, and within-session switching-ratio analyses. (DOCX)
