# Supplementary material for: Continued post-weaning social isolation is associated with persistent, sex-specific changes in sociability and social interaction dynamics in mice
Source: PLoS One. 2026 Oct 26;21(10):e0361386. doi: 10.1371/journal.pone.0361386 (PMC13704843; doi:10.1371/journal.pone.0361386)
Supplement: Supplementary file 1 [file pone.0361386.s002.docx]

**S1 Table.** Primary linear mixed-effects model (LMM) results and complementary RM-ANOVA omnibus results for all behavioral outcomes.

All LMMs included stage × sex × housing fixed effects (sum-to-zero coding) and random intercepts for four-mouse group (cage) and analysis unit nested within cage (mouse for locomotor activity, entropy, and switching; undirected dyad for inter-mouse distance; directed focal–partner dyad for encounter count and contact duration). Outcomes were standardized within measure and phase; variance components are on this standardized scale. Values undefined by construction were treated as missing (light-phase switching ratio coded −1, n = 18; light-phase entropy without an initiated contact, n = 9; light-phase mean contact duration without an initiated encounter, n = 12; all j-SI).

S1A. Random effects and cage likelihood-ratio tests.

| **Fig** | **Measure** | **Phase** | **N obs** | **Cages** | **Units** | **Cage var** | **Unit var** | **Residual var** | **Cage ICC** | **Cage LRT χ²(1)** | **p** |
| --- | --- | --- | --- | --- | --- | --- | --- | --- | --- | --- | --- |
| 1B | Locomotor activity | Light | 288 | 24 | 96 | 0.102 | 0.028 | 0.441 | 0.179 | 10.76 | <0.001 |
| 1C | Locomotor activity | Dark | 288 | 24 | 96 | 0.365 | 0.043 | 0.346 | 0.484 | 50.10 | <0.001 |
| 1D | Inter-mouse distance | Light | 432 | 24 | 144 | 0.251 | 0.000 | 0.369 | 0.405 | 100.62 | <0.001 |
| 1E | Inter-mouse distance | Dark | 432 | 24 | 144 | 0.321 | 0.009 | 0.273 | 0.531 | 117.96 | <0.001 |
| 2A | Social encounter count | Light | 864 | 24 | 288 | 0.193 | 0.091 | 0.551 | 0.231 | 80.53 | <0.001 |
| 2B | Social encounter count | Dark | 864 | 24 | 288 | 0.192 | 0.067 | 0.334 | 0.324 | 123.56 | <0.001 |
| 2C | Mean contact duration per initiated encounter | Light | 852 | 24 | 288 | 0.091 | 0.000 | 0.815 | 0.100 | 33.44 | <0.001 |
| 2D | Mean contact duration per initiated encounter | Dark | 864 | 24 | 288 | 0.283 | 0.043 | 0.621 | 0.298 | 129.02 | <0.001 |
| 3A | Interaction-partner entropy | Light | 279 | 24 | 96 | 0.498 | 0.001 | 0.345 | 0.590 | 64.70 | <0.001 |
| 3B | Interaction-partner entropy | Dark | 288 | 24 | 96 | 0.186 | 0.000 | 0.572 | 0.246 | 27.05 | <0.001 |
| 3C | Switching ratio | Light | 270 | 24 | 95 | 0.045 | 0.000 | 0.919 | 0.046 | 0.57 | 0.226 |
| 3D | Switching ratio | Dark | 288 | 24 | 96 | 0.342 | 0.000 | 0.624 | 0.354 | 40.83 | <0.001 |

S1B. Fixed-effect Wald tests (REML), shown as χ² (p). df = 2 for terms involving stage and 1 otherwise.

| **Fig** | **Phase** | **Stage** | **Sex** | **Housing** | **Stage × Sex** | **Stage × Housing** | **Sex × Housing** | **Stage × Sex × Housing** |
| --- | --- | --- | --- | --- | --- | --- | --- | --- |
| 1B | Light | 51.51 (<0.001) | 0.10 (0.746) | 50.47 (<0.001) | 4.67 (0.097) | 58.92 (<0.001) | 0.22 (0.641) | 0.61 (0.739) |
| 1C | Dark | 26.17 (<0.001) | 0.06 (0.812) | 12.34 (<0.001) | 14.94 (<0.001) | 36.13 (<0.001) | 1.05 (0.306) | 9.04 (0.011) |
| 1D | Light | 63.92 (<0.001) | 0.90 (0.342) | 27.19 (<0.001) | 22.80 (<0.001) | 1.52 (0.468) | 0.09 (0.761) | 27.47 (<0.001) |
| 1E | Dark | 90.44 (<0.001) | 0.65 (0.421) | 24.77 (<0.001) | 2.41 (0.299) | 6.24 (0.044) | 1.33 (0.248) | 26.73 (<0.001) |
| 2A | Light | 140.25 (<0.001) | 0.12 (0.730) | 6.99 (0.008) | 9.66 (0.008) | 36.85 (<0.001) | 0.07 (0.789) | 1.43 (0.488) |
| 2B | Dark | 491.20 (<0.001) | 0.21 (0.646) | 21.15 (<0.001) | 23.14 (<0.001) | 87.94 (<0.001) | 0.31 (0.576) | 10.51 (0.005) |
| 2C | Light | 1.35 (0.510) | 0.33 (0.565) | 21.63 (<0.001) | 3.06 (0.217) | 3.57 (0.167) | 1.57 (0.210) | 6.30 (0.043) |
| 2D | Dark | 14.04 (<0.001) | 0.41 (0.522) | 2.44 (0.118) | 38.92 (<0.001) | 3.04 (0.218) | 1.89 (0.169) | 9.67 (0.008) |
| 3A | Light | 5.86 (0.053) | 2.64 (0.104) | 10.70 (0.001) | 7.41 (0.025) | 7.25 (0.027) | 1.03 (0.309) | 4.13 (0.127) |
| 3B | Dark | 8.75 (0.013) | 3.44 (0.064) | 18.35 (<0.001) | 17.18 (<0.001) | 3.51 (0.173) | 2.32 (0.128) | 6.53 (0.038) |
| 3C | Light | 10.86 (0.004) | 0.00 (0.999) | 0.22 (0.639) | 0.45 (0.797) | 11.66 (0.003) | 0.38 (0.540) | 2.81 (0.245) |
| 3D | Dark | 0.70 (0.704) | 0.00 (0.951) | 0.15 (0.700) | 24.48 (<0.001) | 9.30 (0.010) | 1.60 (0.206) | 8.63 (0.013) |

S1C. Complementary three-way RM-ANOVA, shown as F (p).

| **Fig** | **Phase** | **N units** | **GG ε** | **Stage** | **Sex** | **Housing** | **Stage × Sex** | **Stage × Housing** | **Sex × Housing** | **Stage × Sex × Housing** |
| --- | --- | --- | --- | --- | --- | --- | --- | --- | --- | --- |
| 1B | Light | 96 | 0.921 † | 25.76 (<0.001) | 0.23 (0.632) | 111.57 (<0.001) | 2.34 (0.104) | 29.46 (<0.001) | 0.48 (0.490) | 0.30 (0.721) |
| 1C | Dark | 96 | 0.967 | 13.09 (<0.001) | 0.19 (0.661) | 41.98 (<0.001) | 7.47 (<0.001) | 18.07 (<0.001) | 3.56 (0.062) | 4.52 (0.012) |
| 1D | Light | 144 | 0.929 † | 29.03 (<0.001) | 4.69 (0.032) | 141.39 (<0.001) | 10.35 (<0.001) | 0.69 (0.493) | 0.48 (0.490) | 12.47 (<0.001) |
| 1E | Dark | 144 | 0.873 † | 45.22 (<0.001) | 3.49 (0.064) | 133.57 (<0.001) | 1.21 (0.297) | 3.12 (0.053) | 7.19 (0.008) | 13.37 (<0.001) |
| 2A | Light | 288 | 0.745 † | 70.13 (<0.001) | 0.70 (0.403) | 41.31 (<0.001) | 4.83 (0.016) | 18.43 (<0.001) | 0.42 (0.516) | 0.72 (0.450) |
| 2B | Dark | 288 | 0.735 † | 245.60 (<0.001) | 1.54 (0.216) | 154.16 (<0.001) | 11.57 (<0.001) | 43.97 (<0.001) | 2.28 (0.132) | 5.25 (0.012) |
| 2C | Light | 277 | 0.947 † | 0.69 (0.497) | 0.88 (0.349) | 71.29 (<0.001) | 1.48 (0.229) | 1.77 (0.173) | 5.05 (0.025) | 2.92 (0.058) |
| 2D | Dark | 288 | 0.994 | 7.02 (<0.001) | 3.06 (0.081) | 18.17 (<0.001) | 19.46 (<0.001) | 1.52 (0.219) | 14.11 (<0.001) | 4.83 (0.008) |
| 3A | Light | 90 | 0.978 | 1.04 (0.356) | 6.65 (0.012) | 27.76 (<0.001) | 2.99 (0.053) | 7.98 (<0.001) | 1.90 (0.172) | 3.27 (0.040) |
| 3B | Dark | 96 | 0.849 † | 4.06 (0.025) | 9.97 (0.002) | 53.17 (<0.001) | 7.97 (0.001) | 1.63 (0.203) | 6.71 (0.011) | 3.03 (0.060) |
| 3C | Light | 85 | 0.801 † | 11.02 (<0.001) | 0.43 (0.512) | 5.80 (0.018) | 1.33 (0.265) | 6.26 (0.005) | 0.05 (0.826) | 1.28 (0.276) |
| 3D | Dark | 96 | 0.720 † | 0.34 (0.642) | 0.01 (0.912) | 0.48 (0.491) | 11.81 (<0.001) | 4.49 (0.023) | 5.15 (0.026) | 4.16 (0.029) |

RM-ANOVA used analysis units with complete data at all three stages, with stage as a within-subject factor and sex and housing as between-subject factors (Type III sums of squares). df: between-subject effects (1, N − 4); within-subject effects (2, 2[N − 4]), multiplied by ε when the Greenhouse–Geisser correction was applied (†, Mauchly p < 0.05). Primary inference is based on the LMMs. Values below 0.001 are reported as <0.001.

Abbreviations: GG, Greenhouse–Geisser; ICC, intraclass correlation; LMM, linear mixed-effects model; LRT, likelihood-ratio test (ML fits; p from the 0.5χ²0 + 0.5χ²1 boundary reference); REML, restricted maximum likelihood; RM-ANOVA, repeated-measures analysis of variance; var, variance.
