# Supplementary material for: Continued post-weaning social isolation is associated with persistent, sex-specific changes in sociability and social interaction dynamics in mice
Source: PLoS One. 2026 Oct 26;21(10):e0361386. doi: 10.1371/journal.pone.0361386 (PMC13704843; doi:10.1371/journal.pone.0361386)
Supplement: Supplementary file 2 [file pone.0361386.s003.docx]

**S2 Table.** Bonferroni-adjusted LMM contrasts between housing conditions and between sexes (Figs 1–3).

Entries are differences in estimated marginal means in original units with Bonferroni-adjusted p values (six comparisons per outcome for each contrast family), shown as difference (p). Developmental-stage contrasts, standard errors, z statistics, and unadjusted p values for all contrasts are provided in S1 Data (LMM_contrasts sheet).

S2A. j-SI − GH within sex and developmental stage.

| **Fig** | **Measure** | **Phase** | **Unit** | **P35 male** | **P35 female** | **P49 male** | **P49 female** | **P63 male** | **P63 female** |
| --- | --- | --- | --- | --- | --- | --- | --- | --- | --- |
| 1B | Locomotor activity | Light | m | +16.4 (1.000) | +27.9 (0.557) | +58.2 (0.001) | +74.0 (<0.001) | +110.6 (<0.001) | +109.3 (<0.001) |
| 1C | Locomotor activity | Dark | m | −10.4 (1.000) | +145.4 (0.086) | +83.3 (0.815) | +155.1 (0.054) | +211.9 (<0.001) | +218.2 (0.001) |
| 1D | Inter-mouse distance | Light | mm | +32.7 (0.021) | +43.0 (0.002) | +29.2 (0.054) | +57.4 (<0.001) | +50.2 (<0.001) | +25.6 (0.188) |
| 1E | Inter-mouse distance | Dark | mm | +17.8 (0.200) | +49.0 (<0.001) | +21.9 (0.054) | +30.3 (0.004) | +27.4 (0.006) | +28.4 (0.009) |
| 2A | Social encounter count | Light | count | −59.1 (0.008) | −62.3 (0.009) | −30.3 (0.602) | −23.5 (1.000) | −20.4 (1.000) | −3.87 (1.000) |
| 2B | Social encounter count | Dark | count | −287.8 (<0.001) | −421.7 (<0.001) | −149.0 (0.185) | −205.1 (0.031) | −147.5 (0.196) | −119.2 (0.623) |
| 2C | Mean contact duration per initiated encounter | Light | s | −1.00 (1.000) | −2.77 (0.006) | −0.978 (1.000) | −3.21 (<0.001) | −3.01 (<0.001) | −2.70 (0.009) |
| 2D | Mean contact duration per initiated encounter | Dark | s | +0.290 (1.000) | −1.79 (0.050) | +0.094 (1.000) | −0.975 (0.900) | −0.630 (1.000) | −1.12 (0.581) |
| 3A | Interaction-partner entropy | Light | bits | −0.379 (0.266) | −0.696 (0.003) | −0.219 (1.000) | −0.611 (0.015) | −0.287 (0.786) | −0.378 (0.360) |
| 3B | Interaction-partner entropy | Dark | bits | −0.111 (0.594) | −0.157 (0.169) | −0.142 (0.208) | −0.214 (0.017) | −0.089 (1.000) | −0.349 (<0.001) |
| 3C | Switching ratio | Light | ratio | −0.154 (0.011) | −0.041 (1.000) | +0.052 (1.000) | +0.083 (0.782) | +0.021 (1.000) | −0.031 (1.000) |
| 3D | Switching ratio | Dark | ratio | −0.106 (0.115) | +0.003 (1.000) | −0.075 (0.595) | +0.053 (1.000) | +0.035 (1.000) | +0.022 (1.000) |

S2B. Male − female within housing condition and developmental stage.

| **Fig** | **Measure** | **Phase** | **Unit** | **P35 GH** | **P35 j-SI** | **P49 GH** | **P49 j-SI** | **P63 GH** | **P63 j-SI** |
| --- | --- | --- | --- | --- | --- | --- | --- | --- | --- |
| 1B | Locomotor activity | Light | m | +16.1 (1.000) | +4.61 (1.000) | +17.8 (1.000) | +2.03 (1.000) | −11.9 (1.000) | −10.6 (1.000) |
| 1C | Locomotor activity | Dark | m | +114.0 (0.188) | −41.8 (1.000) | −23.9 (1.000) | −95.7 (0.736) | −0.359 (1.000) | −6.68 (1.000) |
| 1D | Inter-mouse distance | Light | mm | −5.31 (1.000) | −15.6 (1.000) | +20.4 (0.328) | −7.79 (1.000) | −29.8 (0.029) | −5.21 (1.000) |
| 1E | Inter-mouse distance | Dark | mm | +11.5 (0.889) | −19.7 (0.206) | +1.51 (1.000) | −6.95 (1.000) | −6.85 (1.000) | −7.79 (1.000) |
| 2A | Social encounter count | Light | count | +2.81 (1.000) | +5.93 (1.000) | −5.03 (1.000) | −11.8 (1.000) | +25.3 (0.880) | +8.80 (1.000) |
| 2B | Social encounter count | Dark | count | −146.2 (0.152) | −12.3 (1.000) | −56.0 (1.000) | +0.083 (1.000) | +54.8 (1.000) | +26.6 (1.000) |
| 2C | Mean contact duration per initiated encounter | Light | s | −0.935 (1.000) | +0.834 (1.000) | −1.04 (0.989) | +1.19 (1.000) | +0.981 (1.000) | +0.664 (1.000) |
| 2D | Mean contact duration per initiated encounter | Dark | s | −1.65 (0.038) | +0.424 (1.000) | +0.402 (1.000) | +1.47 (0.225) | +0.276 (1.000) | +0.771 (1.000) |
| 3A | Interaction-partner entropy | Light | bits | +0.035 (1.000) | +0.352 (0.567) | −0.077 (1.000) | +0.315 (0.823) | +0.281 (0.698) | +0.372 (0.472) |
| 3B | Interaction-partner entropy | Dark | bits | −0.002 (1.000) | +0.044 (1.000) | −0.013 (1.000) | +0.059 (1.000) | +0.056 (1.000) | +0.316 (<0.001) |
| 3C | Switching ratio | Light | ratio | +0.038 (1.000) | −0.075 (1.000) | +0.024 (1.000) | −0.007 (1.000) | −0.016 (1.000) | +0.037 (1.000) |
| 3D | Switching ratio | Dark | ratio | +0.013 (1.000) | −0.096 (0.337) | +0.035 (1.000) | −0.093 (0.383) | +0.070 (0.636) | +0.083 (0.591) |

Contrasts are Wald z tests on estimated marginal means from the LMMs in S1 Table. Positive values indicate higher values in j-SI (S2A) or in males (S2B). Values below 0.001 are reported as <0.001.

Abbreviations: GH, group-housed; j-SI, juvenile social isolation; LMM, linear mixed-effects model; P35, P49, and P63, postnatal days 35, 49, and 63.
