# Supplementary material for: Continued post-weaning social isolation is associated with persistent, sex-specific changes in sociability and social interaction dynamics in mice
Source: PLoS One. 2026 Oct 26;21(10):e0361386. doi: 10.1371/journal.pone.0361386 (PMC13704843; doi:10.1371/journal.pone.0361386)
Supplement: Supplementary file 3 [file pone.0361386.s004.docx]

**S3 Table.** Exploratory light-phase directionality, partner-specificity, and within-session switching-ratio analyses.

All LMMs included stage × sex × housing fixed effects and random intercepts for four-mouse group and mouse nested within group; effects are shown as Wald χ² (p). These analyses are exploratory and were not corrected for multiple comparisons. Complete records are provided in S1 Data.

S3A. Approach versus receive. For each mouse, A and R are 20-mm initiated-approach and received-event counts; signed index = (A − R)/(A + R) and absolute imbalance = |A − R|/(A + R); duration indices use total 30-mm approach and receive durations.

| **Housing** | **N obs** | **Signed count index** | **Absolute count imbalance** | **Signed duration index** | **Absolute duration imbalance** |
| --- | --- | --- | --- | --- | --- |
| GH | 168 | -0.001 ± 0.019 | 0.194 ± 0.011 | 0.007 ± 0.024 | 0.241 ± 0.015 |
| j-SI | 114 | 0.058 ± 0.044 | 0.365 ± 0.027 | 0.052 ± 0.034 | 0.302 ± 0.021 |

| **Outcome** | **Intercept** | **Stage** | **Sex** | **Housing** | **Stage × Sex** | **Stage × Housing** | **Sex × Housing** | **Stage × Sex × Housing** |
| --- | --- | --- | --- | --- | --- | --- | --- | --- |
| Signed count index | 1.36 (0.243) | 0.24 (0.885) | 1.09 (0.296) | 1.41 (0.235) | 0.56 (0.754) | 0.08 (0.961) | 0.86 (0.353) | 0.74 (0.691) |
| Absolute count imbalance | 124.58 (<0.001) | 0.52 (0.770) | 0.83 (0.364) | 12.56 (<0.001) | 1.83 (0.401) | 2.58 (0.276) | 1.21 (0.271) | 1.42 (0.493) |
| Signed duration index | 1.52 (0.217) | 0.29 (0.865) | 0.00 (0.989) | 1.04 (0.308) | 0.10 (0.952) | 0.59 (0.744) | 0.22 (0.641) | 0.01 (0.995) |
| Absolute duration imbalance | 230.50 (<0.001) | 1.07 (0.585) | 1.34 (0.247) | 2.81 (0.093) | 0.76 (0.683) | 17.01 (<0.001) | 0.60 (0.440) | 4.50 (0.106) |

S3B. Partner concentration and preferred-partner persistence. Maximum partner share is the largest fraction of a mouse’s 20-mm initiated events directed to one of its three partners; the Herfindahl index is the sum of squared partner shares (1/3, even; 1, complete concentration).

| **Housing** | **N obs** | **Maximum partner share** | **Herfindahl index** |
| --- | --- | --- | --- |
| GH | 168 | 0.484 ± 0.008 | 0.396 ± 0.007 |
| j-SI | 111 | 0.637 ± 0.020 | 0.557 ± 0.022 |

| **Outcome** | **Intercept** | **Stage** | **Sex** | **Housing** | **Stage × Sex** | **Stage × Housing** | **Sex × Housing** | **Stage × Sex × Housing** |
| --- | --- | --- | --- | --- | --- | --- | --- | --- |
| Maximum partner share | 623.75 (<0.001) | 4.47 (0.107) | 1.86 (0.173) | 13.27 (<0.001) | 5.82 (0.055) | 7.46 (0.024) | 0.58 (0.447) | 3.67 (0.160) |
| Herfindahl index | 337.36 (<0.001) | 4.55 (0.103) | 2.40 (0.122) | 11.15 (<0.001) | 5.60 (0.061) | 6.35 (0.042) | 0.90 (0.342) | 3.05 (0.217) |

| **Sex** | **Same partner P35–P49: GH vs j-SI (Fisher p)** | **Same partner P49–P63** | **Same partner at all stages** |
| --- | --- | --- | --- |
| male | 15/32 vs 5/18 (0.237) | 11/30 vs 9/18 (0.385) | 7/30 vs 2/18 (0.451) |
| female | 11/24 vs 9/14 (0.328) | 11/23 vs 10/14 (0.191) | 7/23 vs 5/13 (0.720) |

The preferred partner was the unique highest-count partner; tied maxima (n = 8) and stages without initiated events were excluded, so denominators are the numbers of mice with a unique preferred partner at every compared stage.

S3C. Within-session switching ratio (six 30-min light-phase bins; twelve 60-min dark-phase bins; source values of −1 treated as missing). Slopes were estimated for mouse-sessions with at least three valid bins; the early contrast is the first light-phase 30-min bin minus the mean of later valid light bins.

| **Marginal quantity** | **N mouse-sessions** | **N mice** | **Estimate** | **SE** | **Wald χ²(1)** | **p** |
| --- | --- | --- | --- | --- | --- | --- |
| Light slope (per h) | 243 | 87 | −0.0702 | 0.0072 | 96.21 | <0.001 |
| Dark slope (per h) | 278 | 95 | −0.0102 | 0.0020 | 24.71 | <0.001 |
| First 30 min − later light bins | 242 | 87 | 0.1387 | 0.0133 | 108.94 | <0.001 |

| **Outcome** | **Intercept** | **Stage** | **Sex** | **Housing** | **Stage × Sex** | **Stage × Housing** | **Sex × Housing** | **Stage × Sex × Housing** |
| --- | --- | --- | --- | --- | --- | --- | --- | --- |
| Light slope (per h) | 96.21 (<0.001) | 8.21 (0.017) | 1.68 (0.196) | 1.84 (0.175) | 6.50 (0.039) | 8.45 (0.015) | 0.08 (0.784) | 5.80 (0.055) |
| Dark slope (per h) | 24.71 (<0.001) | 1.33 (0.515) | 0.09 (0.770) | 0.20 (0.653) | 1.95 (0.377) | 7.53 (0.023) | 0.13 (0.718) | 1.28 (0.528) |
| First 30 min − later light bins | 108.94 (<0.001) | 4.93 (0.085) | 0.13 (0.724) | 6.23 (0.013) | 3.35 (0.187) | 4.89 (0.087) | 1.90 (0.168) | 7.02 (0.030) |

Values below 0.001 are reported as <0.001.

Abbreviations: GH, group-housed; j-SI, juvenile social isolation; LMM, linear mixed-effects model; N obs, number of mouse-by-stage observations; SE, standard error; SEM, standard error of the mean (descriptive values are mean ± SEM).
