# Supplementary material for: Continued post-weaning social isolation is associated with persistent, sex-specific changes in sociability and social interaction dynamics in mice
Source: PLoS One. 2026 Oct 26;21(10):e0361386. doi: 10.1371/journal.pone.0361386 (PMC13704843; doi:10.1371/journal.pone.0361386)
Supplement: Supplementary file 4 [file pone.0361386.s006.docx]

1. Please ensure that your manuscript meets PLOS ONE's style requirements, including those for file naming. The PLOS ONE style templates can be found at

https://journals.plos.org/plosone/s/file?id=wjVg/PLOSOne_formatting_sample_main_body.pdf and

https://journals.plos.org/plosone/s/file?id=ba62/PLOSOne_formatting_sample_title_authors_affiliations.pdf

**Response: Thank you. We have checked the manuscript against the PLOS ONE style templates for the main body and for the title/authors/affiliations page, and the manuscript files will be reformatted and renamed to comply with the PLOS ONE requirements at resubmission. This is a file-level requirement and is not reflected in the manuscript text itself.**

2. Please note that PLOS One has specific guidelines on code sharing for submissions in which author-generated code underpins the findings in the manuscript. In these cases, we expect all author-generated code to be made available without restrictions upon publication of the work. Please review our guidelines at https://journals.plos.org/plosone/s/materials-and-software-sharing#loc-sharing-code and ensure that your code is shared in a way that follows best practice and facilitates reproducibility and reuse.

**Response:** Thank you for this guidance. All author-generated code used in this study — the in-house Python routines for tracking post-processing and for calculation of locomotor activity, inter-mouse distance, social encounter counts, contact duration, entropy, and switching ratio — has been deposited on Zenodo (DOI: 10.5281/zenodo.21916352) and is available without restriction. A statement to this effect has been added to the Data availability section. (The repository DOI, 10.5281/zenodo.21916352, has now been inserted into the Data availability section.)

3. To comply with PLOS One submissions requirements, in your Methods section, please provide additional information regarding the experiments involving animals and ensure you have included details on (1) methods of sacrifice, (2) methods of anesthesia and/or analgesia, and (3) efforts to alleviate suffering.

**Response:** Thank you. We have added the requested information to the Methods (Mice and housing conditions). The revised text now states the light/dark cycle and husbandry conditions, that marker attachment — the only invasive procedure — was performed under isoflurane anesthesia (1.5–2%), that no analgesia was required because no surgical or otherwise painful procedure was performed, the efforts made to minimize animal number and distress, the daily health monitoring and absence of humane endpoints, and the method of euthanasia at the end of the experimental period.

4. We note that the grant information you provided in the ‘Funding Information’ and ‘Financial Disclosure’ sections do not match.

When you resubmit, please ensure that you provide the correct grant numbers for the awards you received for your study in the ‘Funding Information’ section.

**Response:** Thank you for noting this discrepancy. The grant numbers listed in the Financial Disclosure are the correct ones, and we will enter these identical numbers in the ‘Funding Information’ section of the online submission form at resubmission so that the two sections match. No change to the manuscript text is required for this point.

5. Thank you for stating the following in the Acknowledgments Section of your manuscript: [We thank the members of the Department of Psychiatry, Nara Medical University, for their technical assistance and support with animal care. This work was supported by The NOVARTIS Foundation for the Promotion of Science, SENSHIN Medical Research Foundation, Mochida Memorial Foundation for Medical and Pharmaceutical Research, Kawano Masanori Memorial Public Interest Incorporated Foundation, Inamori Foundation, Senri Life Science Foundation, Astellas Foundation for Research on Metabolic Disorders, Naito Foundation, and Takeda Medical Research Grants; KAKENHI [Grant-in-Aid for Scientific Research numbers 20K15935 and 24K10684 and Grant-in-Aid for Transformative Research Areas number 21H05698 to KY and JP24K02386 to MM]; the Japan Agency for Medical Research and Development (AMED; grant numbers 22wm0525010h0002 to KY and 24gm1910004h0002, 24gm1510009s0103, 25wm0625510s0102, and JP25wm0625126 to MM); and Moonshot R&D (grant number JPMJMS239F-1-2 to MM). The funders had no role in the study design, data collection, data analysis, data interpretation, writing of the report, or decision to submit the article for publication.]

We note that you have provided funding information that is not currently declared in your Funding Statement. However, funding information should not appear in the Acknowledgments section or other areas of your manuscript. We will only publish funding information present in the Funding Statement section of the online submission form.

Please remove any funding-related text from the manuscript and let us know how you would like to update your Funding Statement. Currently, your Funding Statement reads as follows: “The authors received no specific funding for this work.”

Please include your amended statements within your cover letter; we will change the online submission form on your behalf.

**Response:** Thank you. All funding-related text has been removed from the Acknowledgements section, which now reads only: “We thank the members of the Department of Psychiatry, Nara Medical University, for their technical assistance and support with animal care.” The complete, amended funding statement is provided in our cover letter for entry into the online submission form on our behalf.

6. In the online submission form, you indicated that [Data are available from the corresponding author upon reasonable request].

All PLOS journals now require all data underlying the findings described in their manuscript to be freely available to other researchers, either 1. In a public repository, 2. Within the manuscript itself, or 3. Uploaded as supplementary information.

This policy applies to all data except where public deposition would breach compliance with the protocol approved by your research ethics board. If your data cannot be made publicly available for ethical or legal reasons (e.g., public availability would compromise patient privacy), please explain your reasons on resubmission and your exemption request will be escalated for approval.

**Response:** Thank you. We have revised the Data availability section so that the data are made freely available rather than provided on request: the processed behavioral data underlying every figure, together with the underlying positional tracking data, are supplied as Supporting Information (S1 Data), and the analysis code is deposited on Zenodo (DOI: 10.5281/zenodo.21916352). No restrictions apply to the availability of data, code, or materials.

7. Please include your full ethics statement in the ‘Methods’ section of your manuscript file. In your statement, please include the full name of the IRB or ethics committee who approved or waived your study, as well as whether or not you obtained informed written or verbal consent. If consent was waived for your study, please include this information in your statement as well.

**Response:** Thank you. The full ethics statement has been added to the Methods (Mice and housing conditions): all experimental procedures were approved by the Institutional Animal Care and Use Committee of Nara Medical University (Approval No. 13588) and were conducted in accordance with its guidelines. Because this study involved only animals and no human participants, informed written or verbal consent was not applicable, and no consent waiver was required.

8. If the reviewer comments include a recommendation to cite specific previously published works, please review and evaluate these publications to determine whether they are relevant and should be cited. There is no requirement to cite these works unless the editor has indicated otherwise.

**Response:** Thank you. We have reviewed the works referred to in the reviewers’ comments and have cited those that we judged directly relevant to the present findings. No citation was added solely because it was mentioned in the review.

Reviewer #1: The report under review “Juvenile social isolation induces long-lasting, sex-specific changes in social behavior and interaction dynamics in mice” examined the impact of post-weaning social isolation on spontaneous social behavior at multiple time points during both the light and dark phases. Overall the study presents some interesting differences across the light cycle that are novel in the field. However, the data presentation and statistical analyses decrease clarity for the reader.

**Response:** We thank the reviewer for the careful and constructive assessment of our manuscript, and in particular for recognizing the novelty of the light/dark-phase differences. We have revised the statistical reporting, unified the terminology, and clarified the data presentation as detailed below.

1. The statistical analyses in the current manuscript as not entirely appropriate. If there are not significant interactions, then the pair-wise comparisons should not be run. Overall, the large number of comparisons make the take-home message of the findings sometimes difficult to focus on. Perhaps the authors should include a chart with the pairwise comparison data and then focus on the trends across time or within developmental time points. For example, there are developmental trends that are consistent across all groups (i.e. a decrease in social encounter count during the dark phase as they animals get older). Overall, the way the data are presented it is hard to focus on the important differences (i.e. main effects of housing; sex differences and shifts in the developmental time course of these behaviors).

**Response:** We agree. The Statistical analysis section now states explicitly that pairwise comparisons were performed only for effects for which the corresponding main effect or interaction reached significance in the RM-ANOVA, and that no post hoc test was run in the absence of a significant effect. To reduce the visual burden of the large number of comparisons, the complete pairwise comparison results have been moved to a supporting file (S1 Table), and the figures and Results text now emphasize the main effects of housing condition, sex differences, and the developmental trajectories that are consistent across groups (for example, the decrease in social encounter counts during the dark phase with age). Figures 1–3 have been regenerated and S1 Table has been completed; all values in S1 Table were recomputed directly from the raw data and match the GraphPad Prism output.

2. The lack of consistent and precise language when referring to the different variables decreases clarity as a reader. For example, sometimes activity is called activity and sometimes it is total distance traveled. But then “distance” is sometimes used as shorthand to refer to inter-mouse distance which is a different variable. Another example of this is night is often used in place of dark phase. Dark phase is more precise and should be used throughout the manuscript.

**Response:** Thank you — this has been corrected throughout. “Night” has been replaced by “dark phase” in every instance in the main text and figure legends; “locomotor activity” is now used consistently for the total distance traveled by an individual mouse; and “inter-mouse distance” is always written in full, with the unqualified term “distance” no longer used for either measure. A sentence defining this terminology has been added to the Methods.

3. The rationale for the discussion of inter-mouse distance along with activity rather than as a measure of social behavior is unclear.

**Response:** We have added the rationale to the Results. Inter-mouse distance is presented alongside locomotor activity because both are derived from the same continuous positional data and jointly describe how animals distributed themselves in space; it is therefore treated as a spatial-proximity index that is strongly constrained by locomotion, whereas social encounter counts and contact duration serve as the primary measures of social interaction.

4. Are the p-values reported for the post-hoc tests the corrected values based on the multiple comparisons? It is not clear.

**Response:** Yes. All post hoc p values reported in the text and figures are Bonferroni-corrected values adjusted for the number of multiple comparisons; uncorrected p values are not reported. This has now been stated explicitly in the Statistical analysis section.

5. In some cases the graphs are put on a larger scale to fit in all the comparisons which make it harder to see the data. When there is a main effect of housing condition (i.e. the pairwise comparisons are significant between GH and j-SI in both sexes at all time points – Fig. 1e, 2b) this could be indicated in the figure legend instead of all the individual brackets. Additionally, the figures should clearly indicate not only the figure legend that the red and green dots represent the j-SI groups whereas the GH animals are in gray.

**Response:** We agree and have revised the figures accordingly: where the pairwise comparisons are significant between GH and j-SI in both sexes at all time points (Fig 1E, Fig 2B), the y-axis was rescaled so that the data are more clearly visible, and the significance of each GH vs. j-SI comparison is annotated directly on the corresponding pair. The colour coding is now stated explicitly in both the figure legend and the figure panels (grey, GH; colour, j-SI: green for males and red for females). During regeneration, every asterisk was re-derived from the raw data; this showed that several Fig 2B p-values in the previous version were inconsistent with the data, and the legend has been corrected accordingly (P49 and P63 males change from *** to ****, and P63 females from * to ***; all comparisons remain significant).

6. Given the much longer time frame of recording during the dark phase (12h vs. 3h), the data for this phase should be broken up into 3h time blocks to allow for more direct comparisons.

**Response:** Thank you for this suggestion. We would like to clarify the rationale behind our recording design. The 3-h light phase and the 12-h dark phase were never intended as duration-matched windows for direct comparison. The dark phase was the primary observation period because mice are nocturnal and exhibit the great majority of their spontaneous social activity at this time, whereas the short light interval immediately preceding lights-off was included only as an ancillary reference. Consistent with this, the light and dark phases were analyzed entirely separately, and at no point were the two unequal-duration windows compared statistically. Because all of our contrasts are made within a given phase—between housing conditions, sexes, and developmental stages—matching the two phases by subdividing the dark phase into 3-h blocks is not necessary for the comparisons we actually perform. Moreover, adding a time-of-night factor to an already three-factor design (developmental stage × sex × housing) would substantially increase the complexity of the figures and the number of post-hoc comparisons without informing the between-condition questions that are the focus of this study. We have therefore retained the 12-h dark phase as a single analysis window and have clarified this rationale in the Methods.

7. It is not clear from the methods whether the animals began the social interaction test 3hr prior to the end of the light phase such that the light cycle was not shifted during the test. I assume this is the case but it should be stated explicitly. They should also report the lights on/off times and whether the animals are on a 12:12 cycle.

**Response:** The reviewer’s assumption is correct, and we have now stated this explicitly. The Methods now report that mice were maintained on a 12:12-h light/dark cycle with lights on and off at fixed times, and that recording always began 3 h before lights off, so that the 3-h light phase and the subsequent 12-h dark phase corresponded to the animals’ regular cycle; the light/dark cycle was not shifted at any point during testing.

8. While the entropy measure is clearly defined in the methods, it is not clear whether this construct has been validated and is conveying novel information. The control groups do not demonstrate a developmental shift in this measure for example. The authors should discuss more about what the entropy and switching is telling us that is novel.

**Response:** Thank you for this important point. We have added a paragraph to the Discussion explaining what entropy and the switching ratio add beyond total contact time and contact frequency — they describe how evenly contacts are distributed across partners and how frequently partners are alternated — and acknowledging explicitly that these measures have not been formally validated against an independent behavioral standard and that GH controls did not show a developmental shift in entropy. We therefore now present them as descriptive indices of partner-selection structure and treat the j-SI group differences as hypothesis-generating.

Reviewer #2: This manuscript investigates the long-term effects of juvenile social isolation on spontaneous social behavior using an automated long-term behavioral monitoring system. The use of longitudinal behavioral assessment in both male and female mice under relatively naturalistic group conditions is an important strength of the study, and the behavioral tracking system is technically impressive.

However, I have several major concerns regarding the experimental design and statistical analysis that substantially limit confidence in the conclusions. In my opinion, these issues cannot be adequately addressed through revision alone.

**Response:** We thank the reviewer for the positive assessment of the longitudinal design and the tracking platform, and for the detailed critique. We address each concern below and have revised the manuscript accordingly; where a concern requires re-analysis or additional experiments, we state this explicitly rather than overstating what the present revision achieves.

My primary concern is the statistical treatment of the social interaction data. The behavioral measurements (e.g., inter-mouse distance, contact frequency, and contact duration) are generated from interactions among animals within the same cage and are therefore not statistically independent. Nevertheless, the analyses appear to treat pairwise interaction data as independent observations. Because individual animals contribute repeatedly to multiple interaction pairs within the same social group, the effective sample size is substantially inflated, leading to pseudoreplication and potentially exaggerated statistical significance. This issue affects the principal findings throughout the manuscript and would require a fundamentally different analytical framework based on appropriate hierarchical or mixed-effects models.

**Response:** We agree with the reviewer and thank them for identifying this important issue. Our original analysis did not fully account for the non-independence of pairwise observations within the same four-mouse test group. We therefore reanalyzed all outcomes using linear mixed-effects models with developmental stage, sex, housing condition, and their interactions as fixed effects; random intercepts were included for the four-mouse test group and for the analysis unit (individual mouse or dyad, as appropriate) nested within test group. Thus, the 24 four-mouse groups, rather than the individual pairwise observations, defined the independent clustering level. After accounting for this hierarchy, the developmental stage x sex x housing interaction remained significant for inter-mouse distance during both the light phase (Wald χ²(2) = 28.17, p < 0.001) and dark phase (χ²(2) = 26.45, p < 0.001), and for social encounter count during the dark phase (χ²(2) = 10.66, p = 0.0049) but not the light phase (χ²(2) = 1.28, p = 0.528). The interaction also remained significant for social contact duration during the light phase (χ²(2) = 8.71, p = 0.0128) and dark phase (χ²(2) = 34.63, p < 0.001). We have revised the Statistical analysis and Results sections accordingly and have replaced the previous statement that pairwise observations were not treated as independent. We also note the limited number of independent four-mouse groups as a limitation and have moderated the interpretation of statistical significance.

A second major concern relates to the experimental design itself. Animals remained socially isolated throughout the entire experimental period until the final behavioral assessment. Consequently, the study cannot distinguish persistent effects of juvenile isolation from the ongoing effects of continuous social isolation. Therefore, conclusions regarding the "long-lasting" consequences of juvenile isolation are not fully supported by the experimental design.

**Response:** We agree that the present design cannot separate the persistent effects of a time-limited juvenile isolation from the effects of continued isolation, because animals remained individually housed until the final assessment. Rather than claim that isolation restricted to the P21–P35 critical window is by itself sufficient, we have reframed the study as a model of continued post-weaning social deprivation and scoped our claims accordingly: “long-lasting” now denotes persistence of the phenotype under ongoing isolation, not irreversibility after resocialization, and we no longer attribute the observed changes to the critical-window manipulation alone. Dissociating the two components would require a resocialization group (isolation restricted to P21–P35 followed by regrouping), as used in prior work, which we identify as the key next experiment. Once the claims are scoped to what a continued-isolation design supports, the interpretation is fully consistent with the data; the Limitations and Discussion have been revised accordingly.

In addition, the comparison between group-housed and isolated animals is confounded by differences in social familiarity. Group-housed mice were tested with continuously familiar cage mates, whereas isolated mice were reunited with littermates only during behavioral testing. This difference represents a potentially important confounding variable for measures of social distance, contact duration, and interaction dynamics, making it difficult to attribute the observed behavioral changes specifically to juvenile social isolation.

**Response:** We agree that group-housed and isolated animals differ in social familiarity at test. We respectfully note, however, that reduced familiarity is not an extraneous nuisance variable that can be dissociated from isolation: by definition, a socially isolated animal lacks the familiar cage-mates that group housing provides, so diminished familiarity is an intrinsic component of the isolation manipulation rather than a separable confound, and holding familiarity constant would entail removing part of the construct under study. We therefore interpret the reported differences as the combined effect of social deprivation and its inherent reduction in familiarity, and have made this explicit in the Limitations. A design testing group-housed animals with unfamiliar conspecifics could formally partition the familiarity component, and we now note this as an option for future work; we do not think it is required to support the present, appropriately scoped conclusions.

Finally, several interpretations in the Discussion are stronger than supported by the presented data, and some statements are not fully consistent with the reported results.

**Response:** We have moderated the Discussion accordingly. The interpretations are now presented as provisional, the entropy and switching findings are framed as hypothesis-generating descriptive indices rather than validated constructs, and causal attribution to juvenile isolation is explicitly deferred pending studies with resocialization, familiarity-matched controls, and cage-level statistical modeling.

Overall, although the experimental approach is interesting and the behavioral monitoring platform is valuable, the major concerns regarding statistical independence, experimental design, and interpretation substantially weaken the reliability of the conclusions. Because these issues are fundamental rather than editorial, I cannot recommend publication of the manuscript in its current form.

**Response:** We are grateful for this frank assessment. We have addressed the statistical-independence concern by performing the cage-clustered mixed-effects reanalysis described above, and we have revised the statistical methods, results, and limitations accordingly. We have also moderated the interpretation and clearly delimited what the present design can and cannot support. The remaining design limitation—distinguishing persistence after resocialization from effects under continued isolation—would require a resocialization control and is now stated explicitly.

**Additional editorial corrections**

**1. The Author contributions statement omitted Natsuko Kashida, duplicated Mamiko Okuda, and used the same initials for two pairs of authors (MT and TY).**

**Response:** We corrected the contribution statement by adding Natsuko Kashida to the experiment and data-analysis contributions, removing the duplicated Mamiko Okuda entries, and spelling out all author names so that Masato Takahashi/Michihiro Toritsuka and Takahiro Yoshihara/Takahira Yamauchi are unambiguous.

**2. Several Discussion citations did not support the associated claims, including the citations for hypersocial behavior and the subsequent citation groups.**

**Response:** We rechecked the Discussion citations against the reference list and revised both citations and wording. Hypersocial/increased social-preference findings are now cited to Rivera-Irizarry et al. and Myers et al. [23,24]; aggression to Biro et al. [22]; brief behavioral-assay methodology to the appropriate assay references [25,26,36,37]; long-term group tracking to Endo et al. [28,29]; and circuit, sex-dependent, and developmental claims to the corresponding mechanistic and developmental references. We also corrected the parallel statement in the Introduction.

**3. The Abstract overstated the consistency of the inter-mouse-distance effect across developmental stages despite the nonsignificant P63 female light-phase comparison.**

**Response:** We softened the Abstract to state that inter-mouse distance was greater across most developmental stages and sex-phase combinations, which is consistent with Fig 1D and the qualified wording in the Discussion.
