# Supplementary material for: Continued post-weaning social isolation is associated with persistent, sex-specific changes in sociability and social interaction dynamics in mice
Source: PLoS One. 2026 Oct 26;21(10):e0361386. doi: 10.1371/journal.pone.0361386 (PMC13704843; doi:10.1371/journal.pone.0361386)
Supplement: Supplementary file 5 [file pone.0361386.s007.docx]

**Response to Reviewers**

Manuscript: “Continued post-weaning social isolation is associated with persistent, sex-specific changes in sociability and social interaction dynamics in mice”

PLOS ONE

We thank the Editor and Reviewers #3 and #4 for their further careful reading and constructive suggestions. We have revised the manuscript accordingly. Below, each comment is reproduced in **bold**, our response follows in plain text, and text newly added or changed in the manuscript is shown in red. All manuscript changes are marked in red in the revised file; a clean copy is also provided.

Additional corrections made during final verification. While cross-checking the manuscript, supporting tables, and S1 Data, we identified and corrected three data-handling issues; each is documented in S1 Data (README and Analysis_notes). (i) Values that are undefined by construction had been entered as numbers in three light-phase outcomes: switching ratio coded −1 by the source pipeline when fewer than two initiated contacts occurred (n = 18 mouse-stage observations), entropy for mice without an initiated contact (n = 9), and mean contact duration for directed dyads without an initiated encounter (n = 12). All were j-SI observations. They are now treated as missing, consistent with the time-course analysis, and the affected LMMs, contrasts, and figures were recalculated. (ii) Tied highest-count partners (n = 8) are now excluded from the preferred-partner persistence analysis, as stated in the Methods. (iii) Inconsistent labels for four mice at P49 in the switching time-course files were harmonized, and the time-course models were refitted. In addition, in response to Reviewer #3’s request that LMMs be reported consistently, all post hoc comparisons and significance annotations in Figs 1–3 are now LMM contrasts (S2 Table and S1 Data), and RM-ANOVA is reported as complementary omnibus results (S1 Table). Because the complete processed data and all model outputs are provided in S1 Data, the supporting tables were consolidated into three tables: S1 Table (primary LMM and complementary RM-ANOVA results; previously S3 Table), S2 Table (housing and sex contrasts), and S3 Table (exploratory directionality, partner-specificity, and time-course analyses; previously S4–S6 Tables). The consequences for the findings are summarized under Reviewer #3, Comment 2.

**Reviewer #3**

**Comment 1. The authors should explicitly mention in the Abstract and highlight in the relevant sections the most important limitation of their study: isolated mice were tested with unfamiliar mice (cage mates separated for over two weeks; therefore unfamiliar animals), whereas group-housed mice were tested with their familiar cage mates.**

***Response.*** We fully agree and thank the reviewer for pressing this point. We had previously described the reunited littermates as “previously familiar,” but we accept that after separation exceeding two weeks—longer than the typical persistence of rodent social recognition memory—the reunited partners should be regarded as **effectively unfamiliar**. We now state this explicitly in the Abstract, the Methods (Mice and housing conditions), and the Limitations, and we frame reduced familiarity as an intrinsic, inseparable component of the isolation manipulation rather than a nuisance variable.

*Abstract (added):* “An important design feature is that isolated mice were reunited at testing with cage mates from which they had been separated for more than two weeks and were therefore effectively unfamiliar, whereas group-housed mice were tested with continuously familiar cage mates; the reported effects therefore reflect social isolation together with its intrinsic reduction in social familiarity.”

*Methods (revised):* “…with whom they had been co-housed only before weaning (until P21). Because these mice had then been separated for more than two weeks before the first test (P35) and for progressively longer before the later tests (P49 and P63)—intervals that exceed the typical duration of rodent social recognition memory—the reunited partners were effectively unfamiliar at testing. In contrast, group-housed mice were tested with cage mates with whom they had remained continuously familiar…”

Limitations (revised, excerpt): “…GH mice interacted with continuously familiar cage mates, whereas j-SI mice were reunited with original littermates after more than two weeks of separation and should be regarded as effectively unfamiliar. Reduced familiarity is therefore inseparable from the isolation manipulation in this design, and the observed differences cannot be attributed to individual housing alone. A familiarity-matched control would require testing both housing groups with unfamiliar conspecifics.”

**Comment 2. The authors should use the statistical approach most appropriate for the longitudinal structure of the data. LMM is more appropriate… The authors should use LMMs as the primary statistical analysis and report the results consistently throughout the statistical methods, Results, figures, and/or supplementary table. Remove the separate “Cage-clustered reanalyses” section, or add the RM-ANOVA results and what distinct question it addresses. In particular, clarify if the cage effect between the two housing conditions was statistically significant in this model.**

Response. We agree. We recovered the cage and analysis-unit identifiers from the archived source files and recalculated all 12 primary longitudinal models. Each LMM includes developmental stage × sex × housing fixed effects, a random intercept for the four-mouse test group, and a nested random intercept for the analysis unit. The analysis includes 24 independent groups (8 male GH, 5 male j-SI, 6 female GH, 5 female j-SI), 96 mice, 144 undirected dyads, and 288 directed dyads. REML is used for variance components and fixed-effect Wald tests; ML and a boundary-adjusted likelihood-ratio test are used for the cage component. LMMs now provide the primary inference throughout, including all post hoc comparisons: the significance annotations in Figs 1–3, the Results, and the figure legends are based on Bonferroni-adjusted contrasts of LMM estimated marginal means (S2 Table; all contrasts in S1 Data). RM-ANOVA is retained as a complementary within-animal developmental analysis, and its omnibus results are reported in S1 Table. Full results and identifiers are supplied in S1 Table and S1 Data.

Cage effect. The cage random intercept was supported in 11 of 12 models (ML likelihood-ratio χ²(1) = 10.76–129.02, all boundary-adjusted p < 0.001); the exception was light-phase switching ratio after undefined values were treated as missing (χ²(1) = 0.57, p = 0.226). The housing fixed effect was significant in 9 of 12 models; the exceptions were dark-phase contact duration (χ²(1) = 2.44, p = 0.118), light-phase switching ratio (χ²(1) = 0.22, p = 0.639), and dark-phase switching ratio (χ²(1) = 0.15, p = 0.700). Thus, housing-condition differences and residual cage-to-cage heterogeneity are statistically distinct and are now reported separately.

Statistical analysis (revised, excerpt): “…Random intercepts were included for four-mouse test group (cage) and analysis unit nested within cage… Variance components and fixed effects were estimated by restricted maximum likelihood (REML); joint fixed-effect terms were evaluated using Wald χ² tests… The cage random intercept was tested by refitting models by maximum likelihood (ML), comparing the full model with a model omitting cage, and using the 0.5χ²0 + 0.5χ²1 boundary reference…”

For reference, the recalculated stage × sex × housing interactions were: locomotor activity, light χ²(2) = 0.61, p = 0.739 and dark χ²(2) = 9.04, p = 0.0109; inter-mouse distance, light χ²(2) = 27.47, p < 0.001 and dark χ²(2) = 26.73, p < 0.001; social encounter count, light χ²(2) = 1.43, p = 0.488 and dark χ²(2) = 10.51, p = 0.0052; mean contact duration, light χ²(2) = 6.30, p = 0.0429 and dark χ²(2) = 9.67, p = 0.0080; entropy, light χ²(2) = 4.13, p = 0.127 and dark χ²(2) = 6.53, p = 0.0382; switching ratio, light χ²(2) = 2.81, p = 0.245 and dark χ²(2) = 8.63, p = 0.0134.

Consequences of LMM-based contrasts. Because the LMM accounts for cage clustering, several differences that had been significant in the RM-ANOVA post hoc tests were not supported by the LMM contrasts, and the text was revised accordingly. In males, lower switching after j-SI is now confined to the light phase at P35 (previously also reported for the dark phase at P35 and P49), and fewer dark-phase encounters are significant only at P35 (previously P35, P49, and P63). Lower interaction-partner entropy after j-SI is now confined to females (light phase at P35 and P49; dark phase at P49 and P63). Several previously reported sex contrasts (e.g., greater dark-phase locomotor activity in GH males than GH females at P35) were not significant. The central findings—greater inter-mouse distance, later-emerging increases in locomotor activity, reduced early encounter counts, and shorter light-phase contact duration after j-SI—were retained. The Abstract, Results, figure legends, and Discussion were updated.

**Comment 3. The statement at Line 432 (“our findings do not support the interpretation that isolated males exhibit enhanced sociability”) and similar statements appear too strong. (a) The data rely on global parameters aggregated over the recording period; sociability was not examined as a function of time within a session. Examining the first 5–10 min after reunion separately from the remainder could distinguish an acute response from overall interaction. (b) Aggression/territoriality is another confound in isolated males. Either perform additional analyses for aggressive responses or temper the interpretation and acknowledge these factors.**

Response. We both tempered the interpretation and performed the additional time-course analysis supported by the archived files. Switching ratio was available in six 30-min light-phase bins and twelve 60-min dark-phase bins. It declined within both phases (marginal slopes: light −0.070/h, SE 0.007, p < 0.001; dark −0.0102/h, SE 0.0020, p < 0.001), and the first 30 min of the light phase exceeded the later-bin mean by 0.139 (SE 0.013, p < 0.001). We added these exploratory results, Fig 4, S3 Table, and the time-binned data. The archive does not resolve the first 5–10 min and contains no ethological aggression labels or suitable fine-action video scoring, so those two requested analyses remain impossible from the available data; the manuscript now states this limitation precisely.

Discussion (revised): “…the exploratory switching time course showed higher switching in the first 30 min followed by a within-session decline. This supports an early-versus-later change in interaction sequencing, while the available 30-min bins cannot resolve the first 5–10 min and no comparable time course was available for approach count, duration, locomotion, or aggression.”

Limitations (revised): “…dorsal-marker tracking cannot directly score biting, tail rattling, chasing, wrestling, dominance, or aggression… only switching ratio was available at subphase resolution, in six 30-min light bins and twelve 60-min dark bins. These data demonstrate an early-to-later decline in switching but cannot resolve the first 5–10 min or time courses for other measures…”

**Comment 4. (MINOR) The term “social behaviors” is overly broad; replace with more specific terms such as “sociability” or “social interaction” as appropriate.**

***Response.*** Done. We have replaced the generic term “social behavior(s)” with “sociability” or “social interaction” throughout, wherever a specific measured construct is meant, retaining the broad term only where we genuinely refer to the general construct (e.g., background statements). We also added a one-sentence operational definition to the Methods.

*Methods (added):* “Throughout, we use ‘sociability’ to denote the tendency to approach and maintain social contact and ‘social interaction’ to denote the measured contact events and their temporal organization; other categories of social behavior (e.g., aggression, territorial, or maternal behavior) were not assessed.”

For consistency with the measured constructs, we updated the title to “…persistent, sex-specific changes in sociability and social interaction dynamics in mice.”

**Reviewer #4**

We thank Reviewer #4 for the generous assessment of the novelty and strengths of the study and for a set of suggestions that have substantially improved the clarity and depth of the manuscript.

**Comment 4.1 (direction of effects). The results would be easier to follow if statements of statistical outcomes were accompanied by the direction of differences among groups (e.g., the direction of sex differences is never stated); whenever post hoc differences are mentioned, say which direction they are in.**

**Response.** We agree and have added the direction of every reported post hoc difference—housing, sex, and developmental-stage contrasts—in the Results and in all figure legends. Housing and sex contrasts are tabulated with signed estimates in S2 Table, and all contrasts, including developmental-stage contrasts, are provided in S1 Data. For example, within GH mice, males had a shorter light-phase inter-mouse distance than females at P63 and a shorter dark-phase mean contact duration than females at P35; at P63, j-SI females had lower dark-phase entropy than j-SI males; and developmental changes are stated as increases or decreases (e.g., within j-SI mice, distance traveled increased from P35 to P49 and from P49 to P63 in both sexes).

Examples (Results, revised): “j-SI mice had fewer light-phase social encounters than GH mice at P35 in both sexes”; “Light-phase mean contact duration per initiated encounter was shorter in j-SI than in GH females at all three stages”; and “The light-phase switching ratio was lower in j-SI than in GH males at P35 and then increased within j-SI males from P35 to P49 and P63.”

**Comment 4.2 (individual identity / partner-specificity). Lower entropy and switching for isolated mice could suggest patterns of association (isolated mice spend more time with specific individuals). Is this correct? Are those individuals distinct in any way? Dominance status was not examined, but could the status of the socially housed individuals matter in patterns of association?**

Response. We recovered the light-phase 20-mm directed partner matrices and performed the requested partner-specificity analysis. j-SI mice directed a larger maximum share of initiated events to one partner than GH mice (descriptive means 0.637 vs 0.484; housing Wald χ²(1) = 13.27, p < 0.001), with the same conclusion from the Herfindahl index (0.557 vs 0.396; χ²(1) = 11.15, p < 0.001). We also tracked the identity of the highest-count partner across stages. Persistence was not consistently greater in j-SI mice; all sex-specific Fisher exact comparisons were non-significant (all p ≥ 0.191); mice whose highest count was tied between partners were excluded from this analysis. Thus, isolation was associated with greater concentration on one partner within a session, but not clear developmental persistence of the same partner. Dominance rank and other partner traits were not measured and cannot be inferred. Full results are in S3 Table and S1 Data.

Discussion/Limitations (revised): “Direct partner-level analysis confirmed that j-SI mice concentrated a larger share of initiated light-phase events on a single partner. However, preferred-partner identity was not consistently more stable across development, and the dataset contains no dominance rank or other partner trait with which to characterize the selected individual.”

**Comment 4.3 (behavior over time / stability within the assay). Given the long recording period, it seems a good opportunity to measure how behavior changes over time. Are the measured behaviors stable over time in the interaction assay, or do they change?**

Response. We agree and have now added the time-course analysis permitted by the archived files. Switching ratio was analyzed over six 30-min light-phase bins and twelve 60-min dark-phase bins, with source values of −1 (undefined because of insufficient interactions) treated as missing. Switching declined within both phases, and the first 30 min was higher than the later light-phase bins. Fig 4, S3 Table, and the full time-binned observations have been added. Because no finer bins or time courses for the other outcomes were present, we do not claim to resolve the first 5–10 min or the stability of every behavioral measure.

**Comment 4.4 (fighting on reunification). Fighting often occurs when isolated mice are placed with conspecifics and is commonly increased in isolated mice. Did fighting occur with reunification?**

***Response.*** We did not formally score fighting. Because the AR-LABO system records the position of a dorsal ID marker and not fine-scale actions, it cannot detect biting, tail rattling, or wrestling, so we cannot quantify aggression directly and do not wish to over-interpret positional proxies. We now state this limitation explicitly (see also Reviewer #3, point 3b) and note that increased locomotor activity and greater inter-mouse distance in isolated males are compatible with heightened aggression/vigilance but are not diagnostic of it; dedicated video-based ethological scoring will be required.

**Comment 4.5 (approach vs. receiving; proximity as affiliation vs. aggression). Approach versus receiving is described in the Methods, but there is no analysis in the Results. Are there differences between approaching and receiving depending on housing? Also, proximity could decrease with affiliation or aggression—is there a reason to interpret proximity as one or the other?**

Response. We recovered the light-phase approach and receive records and completed the requested analysis. Each approach by one mouse is the corresponding receive event for its partner, so four-mouse-group totals necessarily balance. The signed individual initiation index showed neither a deviation from zero (p = 0.243) nor a housing effect (p = 0.235). In contrast, the absolute individual imbalance was greater in j-SI mice than GH mice (0.365 vs 0.194; housing χ²(1) = 12.56, p < 0.001), indicating stronger specialization into relatively more initiating or more receiving roles without a population-wide shift in direction. These results are reported in S3 Table and S1 Data. We also clarify that greater spacing is valence-ambiguous and can reflect reduced affiliation, avoidance, vigilance, or territoriality.

Discussion (revised): “Greater inter-mouse distance is interpreted as reduced spatial proximity, but proximity is ambiguous in valence: it may reflect diminished affiliation, active avoidance, vigilance, or territorial spacing toward a less familiar conspecific. Positional data alone cannot distinguish these possibilities.”

**Comment 4.6 (more Discussion context: entropy/switching; light vs. dark cycle). Provide more context for findings: explain the concept of partner switching / entropy in relation to the outcomes more deeply, and explain what the behavioral differences between light and dark cycles could mean.**

***Response.*** We have expanded the Discussion in both respects.

Discussion (revised, entropy/switching): “…an animal can maintain the same total contact time while distributing it evenly across partners (high entropy) or unevenly (low entropy), and can alternate partners frequently (high switching) or persist with one partner (low switching). The observed reductions therefore indicate a more uneven distribution of contacts and less frequent partner change, not necessarily contact with fewer partners.”

*Discussion (added, light vs. dark):* “…dark-phase measures reflect behavior during peak activity, when motivational and circadian drives are highest. The stage × sex × housing interactions detected during the dark phase, but not the light phase, for locomotor activity, encounter counts, and switching ratio… are consistent with isolation-related differences being most readily expressed when animals are most active… the phase-dependent pattern suggests that the behavioral consequences of juvenile isolation are state-dependent and most evident during periods of high spontaneous activity.”

**Comment 4.7 (intensive social sessions may alter trajectory). It is true that there are several intensive periods of social activity at P35, P49, and P63 that could also alter the trajectory of behavior. Could this point be added?**

***Response.*** Yes—this is an important qualification of the longitudinal design, and we have added it.

*Discussion/Limitations (added):* “…the longitudinal design itself includes three prolonged periods of intensive social interaction (at P35, P49, and P63), during which otherwise isolated mice experienced 15 h of group housing. These repeated re-exposures could themselves influence the developmental trajectory of social behavior—for example, by providing intermittent social experience or by cumulative habituation to the test context—so the later time points reflect not only continued isolation but also the effect of prior testing sessions.”

Data and reproducibility additions. The revised S1 Data workbook now includes the cage/unit identifiers, approach–receive records, partner matrices and selectivity summaries, switching time bins, and all LMM outputs. The analysis code and derived CSV outputs are supplied as a separate reproducibility package, including the reanalysis code for the corrected data, LMM contrasts (new LMM_contrasts and LMM_EMMs sheets), and RM-ANOVA (new RM_ANOVA sheet; S1 Table).

We are grateful to both reviewers; we believe these revisions have materially strengthened the manuscript and hope it is now suitable for publication in PLOS ONE.
