# Supplementary material for: Continued post-weaning social isolation is associated with persistent, sex-specific changes in sociability and social interaction dynamics in mice
Source: PLoS One. 2026 Oct 26;21(10):e0361386. doi: 10.1371/journal.pone.0361386 (PMC13704843; doi:10.1371/journal.pone.0361386)
Supplement: Peer review history

# Peer review: Decision Letter 0

**DOI:** 10.1371/journal.pone.0361386.r001

**Authors:** Laurence Coutellier

3 Aug 2026

Dear Dr. Yamamuro,

Thank you for submitting your manuscript to PLOS ONE. After careful consideration, we feel that it has merit but does not fully meet PLOS ONE’s publication criteria as it currently stands. Therefore, we invite you to submit a revised version of the manuscript that addresses the points raised during the review process.

Please submit your revised manuscript by Oct 02 2026 11:59PM. If you will need more time than this to complete your revisions, please reply to this message or contact the journal office at plosone@plos.org. When you're ready to submit your revision, log on to https://www.editorialmanager.com/pone/ and select the 'Submissions Needing Revision' folder to locate your manuscript file.

- A letter that responds to each point raised by the academic editor and reviewer(s). You should upload this letter as a separate file labeled 'Response to Reviewers'.
- A marked-up copy of your manuscript that highlights changes made to the original version. You should upload this as a separate file labeled 'Revised Manuscript with Track Changes'.
- An unmarked version of your revised paper without tracked changes. You should upload this as a separate file labeled 'Manuscript'.

If you would like to make changes to your financial disclosure, please include your updated statement in your cover letter. Guidelines for resubmitting your figure files are available below the reviewer comments at the end of this letter.

If applicable, we recommend that you deposit your laboratory protocols in protocols.io to enhance the reproducibility of your results. Protocols.io assigns your protocol its own identifier (DOI) so that it can be cited independently in the future. For instructions see: https://journals.plos.org/plosone/s/submission-guidelines#loc-laboratory-protocols. Additionally, PLOS ONE offers an option for publishing peer-reviewed Lab Protocol articles, which describe protocols hosted on protocols.io. Read more information on sharing protocols at https://plos.org/protocols?utm_medium=editorial-email&utm_source=authorletters&utm_campaign=protocols.

As the corresponding author, your ORCID iD is verified in the submission system and will appear in the published article. PLOS supports the use of ORCID, and we encourage all coauthors to register for an ORCID iD and use it as well. Please encourage your coauthors to verify their ORCID iD within the submission system before final acceptance, as unverified ORCID iDs will not appear in the published article. *Only*  the individual author can complete the verification step; PLOS staff *cannot*  verify ORCID iDs on behalf of authors.

We look forward to receiving your revised manuscript.

Kind regards,

Laurence Coutellier, PhD

Academic Editor

PLOS One

Journal Requirements:

When submitting your revision, we need you to address these additional requirements.

1. Please ensure that your manuscript meets PLOS ONE's style requirements, including those for file naming. The PLOS ONE style templates can be found at

https://journals.plos.org/plosone/s/file?id=wjVg/PLOSOne_formatting_sample_main_body.pdf and

https://journals.plos.org/plosone/s/file?id=ba62/PLOSOne_formatting_sample_title_authors_affiliations.pdf

2. Please note that PLOS One has specific guidelines on code sharing for submissions in which author-generated code underpins the findings in the manuscript. In these cases, we expect all author-generated code to be made available without restrictions upon publication of the work. Please review our guidelines at https://journals.plos.org/plosone/s/materials-and-software-sharing#loc-sharing-code and ensure that your code is shared in a way that follows best practice and facilitates reproducibility and reuse.

3. To comply with PLOS One submissions requirements, in your Methods section, please provide additional information regarding the experiments involving animals and ensure you have included details on (1) methods of sacrifice, (2) methods of anesthesia and/or analgesia, and (3) efforts to alleviate suffering.

4. We note that the grant information you provided in the ‘Funding Information’ and ‘Financial Disclosure’ sections do not match.

When you resubmit, please ensure that you provide the correct grant numbers for the awards you received for your study in the ‘Funding Information’ section.

5. Thank you for stating the following in the Acknowledgments Section of your manuscript: [We thank the members of the Department of Psychiatry, Nara Medical University, for their technical assistance and support with animal care. This work was supported by The NOVARTIS Foundation for the Promotion of Science, SENSHIN Medical Research Foundation, Mochida Memorial Foundation for Medical and Pharmaceutical Research, Kawano Masanori Memorial Public Interest Incorporated Foundation, Inamori Foundation, Senri Life Science Foundation, Astellas Foundation for Research on Metabolic Disorders, Naito Foundation, and Takeda Medical Research Grants; KAKENHI [Grant-in-Aid for Scientific Research numbers 20K15935 and 24K10684 and Grant-in-Aid for Transformative Research Areas number 21H05698 to KY and JP24K02386 to MM]; the Japan Agency for Medical Research and Development (AMED; grant numbers 22wm0525010h0002 to KY and 24gm1910004h0002, 24gm1510009s0103, 25wm0625510s0102, and JP25wm0625126 to MM); and Moonshot R&D (grant number JPMJMS239F-1-2 to MM). The funders had no role in the study design, data collection, data analysis, data interpretation, writing of the report, or decision to submit the article for publication.]

We note that you have provided funding information that is not currently declared in your Funding Statement. However, funding information should not appear in the Acknowledgments section or other areas of your manuscript. We will only publish funding information present in the Funding Statement section of the online submission form.

Please remove any funding-related text from the manuscript and let us know how you would like to update your Funding Statement. Currently, your Funding Statement reads as follows: “The authors received no specific funding for this work.”

Please include your amended statements within your cover letter; we will change the online submission form on your behalf.

6. In the online submission form, you indicated that [Data are available from the corresponding author upon reasonable request].

All PLOS journals now require all data underlying the findings described in their manuscript to be freely available to other researchers, either 1. In a public repository, 2. Within the manuscript itself, or 3. Uploaded as supplementary information.

This policy applies to all data except where public deposition would breach compliance with the protocol approved by your research ethics board. If your data cannot be made publicly available for ethical or legal reasons (e.g., public availability would compromise patient privacy), please explain your reasons on resubmission and your exemption request will be escalated for approval.

7.  Please include your full ethics statement in the ‘Methods’ section of your manuscript file. In your statement, please include the full name of the IRB or ethics committee who approved or waived your study, as well as whether or not you obtained informed written or verbal consent. If consent was waived for your study, please include this information in your statement as well.

8. If the reviewer comments include a recommendation to cite specific previously published works, please review and evaluate these publications to determine whether they are relevant and should be cited. There is no requirement to cite these works unless the editor has indicated otherwise.

[Note: HTML markup is below. Please do not edit.]

Reviewers' comments:

Reviewer's Responses to Questions

**Comments to the Author**

1. Is the manuscript technically sound, and do the data support the conclusions?

Reviewer #1: Yes

Reviewer #2: No

**********

2. Has the statistical analysis been performed appropriately and rigorously?

Reviewer #1: Yes

Reviewer #2: No

**********

3. Have the authors made all data underlying the findings in their manuscript fully available?

The PLOS Data policy

Reviewer #1: Yes

Reviewer #2: Yes

**********

4. Is the manuscript presented in an intelligible fashion and written in standard English?

Reviewer #1: No

Reviewer #2: Yes

**********

Reviewer #1: The report under review “Juvenile social isolation induces long-lasting, sex-specific changes in social behavior and interaction dynamics in mice” examined the impact of post-weaning social isolation on spontaneous social behavior at multiple time points during both the light and dark phases. Overall the study presents some interesting differences across the light cycle that are novel in the field. However, the data presentation and statistical analyses decrease clarity for the reader.

1. The statistical analyses in the current manuscript as not entirely appropriate. If there are not significant interactions, then the pair-wise comparisons should not be run. Overall, the large number of comparisons make the take-home message of the findings sometimes difficult to focus on. Perhaps the authors should include a chart with the pairwise comparison data and then focus on the trends across time or within developmental time points. For example, there are developmental trends that are consistent across all groups (i.e. a decrease in social encounter count during the dark phase as they animals get older). Overall, the way the data are presented it is hard to focus on the important differences (i.e. main effects of housing; sex differences and shifts in the developmental time course of these behaviors).

2. The lack of consistent and precise language when referring to the different variables decreases clarity as a reader. For example, sometimes activity is called activity and sometimes it is total distance traveled. But then “distance” is sometimes used as shorthand to refer to inter-mouse distance which is a different variable. Another example of this is night is often used in place of dark phase. Dark phase is more precise and should be used throughout the manuscript.

3. The rationale for the discussion of inter-mouse distance along with activity rather than as a measure of social behavior is unclear.

4. Are the p-values reported for the post-hoc tests the corrected values based on the multiple comparisons? It is not clear.

5. In some cases the graphs are put on a larger scale to fit in all the comparisons which make it harder to see the data. When there is a main effect of housing condition (i.e. the pairwise comparisons are significant between GH and j-SI in both sexes at all time points – Fig. 1e, 2b) this could be indicated in the figure legend instead of all the individual brackets. Additionally, the figures should clearly indicate not only the figure legend that the red and green dots represent the j-SI groups whereas the GH animals are in gray.

6. Given the much longer time frame of recording during the dark phase (12h vs. 3h), the data for this phase should be broken up into 3h time blocks to allow for more direct comparisons.

7. It is not clear from the methods whether the animals began the social interaction test 3hr prior to the end of the light phase such that the light cycle was not shifted during the test. I assume this is the case but it should be stated explicitly. They should also report the lights on/off times and whether the animals are on a 12:12 cycle.

8. While the entropy measure is clearly defined in the methods, it is not clear whether this construct has been validated and is conveying novel information. The control groups do not demonstrate a developmental shift in this measure for example. The authors should discuss more about what the entropy and switching is telling us that is novel.

Reviewer #2: This manuscript investigates the long-term effects of juvenile social isolation on spontaneous social behavior using an automated long-term behavioral monitoring system. The use of longitudinal behavioral assessment in both male and female mice under relatively naturalistic group conditions is an important strength of the study, and the behavioral tracking system is technically impressive.

However, I have several major concerns regarding the experimental design and statistical analysis that substantially limit confidence in the conclusions. In my opinion, these issues cannot be adequately addressed through revision alone.

My primary concern is the statistical treatment of the social interaction data. The behavioral measurements (e.g., inter-mouse distance, contact frequency, and contact duration) are generated from interactions among animals within the same cage and are therefore not statistically independent. Nevertheless, the analyses appear to treat pairwise interaction data as independent observations. Because individual animals contribute repeatedly to multiple interaction pairs within the same social group, the effective sample size is substantially inflated, leading to pseudoreplication and potentially exaggerated statistical significance. This issue affects the principal findings throughout the manuscript and would require a fundamentally different analytical framework based on appropriate hierarchical or mixed-effects models.

A second major concern relates to the experimental design itself. Animals remained socially isolated throughout the entire experimental period until the final behavioral assessment. Consequently, the study cannot distinguish persistent effects of juvenile isolation from the ongoing effects of continuous social isolation. Therefore, conclusions regarding the "long-lasting" consequences of juvenile isolation are not fully supported by the experimental design.

In addition, the comparison between group-housed and isolated animals is confounded by differences in social familiarity. Group-housed mice were tested with continuously familiar cage mates, whereas isolated mice were reunited with littermates only during behavioral testing. This difference represents a potentially important confounding variable for measures of social distance, contact duration, and interaction dynamics, making it difficult to attribute the observed behavioral changes specifically to juvenile social isolation.

Finally, several interpretations in the Discussion are stronger than supported by the presented data, and some statements are not fully consistent with the reported results.

Overall, although the experimental approach is interesting and the behavioral monitoring platform is valuable, the major concerns regarding statistical independence, experimental design, and interpretation substantially weaken the reliability of the conclusions. Because these issues are fundamental rather than editorial, I cannot recommend publication of the manuscript in its current form.

**********

what does this mean?). If published, this will include your full peer review and any attached files.

If you choose “no”, your identity will remain anonymous but your review may still be made public.

**Do you want your identity to be public for this peer review?**  For information about this choice, including consent withdrawal, please see our Privacy Policy

Reviewer #1: No

Reviewer #2: No

**********

[NOTE: If reviewer comments were submitted as an attachment file, they will be attached to this email and accessible via the submission site. Please log into your account, locate the manuscript record, and check for the action link "View Attachments". If this link does not appear, there are no attachment files.]

To ensure your figures meet our technical requirements, please review our figure guidelines: https://journals.plos.org/plosone/s/figures

You may also use PLOS’s free figure tool, NAAS, to help you prepare publication quality figures: https://journals.plos.org/plosone/s/figures#loc-tools-for-figure-preparation.

NAAS will assess whether your figures meet our technical requirements by comparing each figure against our figure specifications.

# Author comment: Author response to Decision Letter 1

**DOI:** 10.1371/journal.pone.0361386.r002

18 Aug 2026

1. Please ensure that your manuscript meets PLOS ONE's style requirements, including those for file naming. The PLOS ONE style templates can be found at

https://journals.plos.org/plosone/s/file?id=wjVg/PLOSOne_formatting_sample_main_body.pdf and

https://journals.plos.org/plosone/s/file?id=ba62/PLOSOne_formatting_sample_title_authors_affiliations.pdf

Response: Thank you. We have checked the manuscript against the PLOS ONE style templates for the main body and for the title/authors/affiliations page, and the manuscript files will be reformatted and renamed to comply with the PLOS ONE requirements at resubmission. This is a file-level requirement and is not reflected in the manuscript text itself.

2. Please note that PLOS One has specific guidelines on code sharing for submissions in which author-generated code underpins the findings in the manuscript. In these cases, we expect all author-generated code to be made available without restrictions upon publication of the work. Please review our guidelines at https://journals.plos.org/plosone/s/materials-and-software-sharing#loc-sharing-code and ensure that your code is shared in a way that follows best practice and facilitates reproducibility and reuse.

Response: Thank you for this guidance. All author-generated code used in this study — the in-house Python routines for tracking post-processing and for calculation of locomotor activity, inter-mouse distance, social encounter counts, contact duration, entropy, and switching ratio — has been deposited on Zenodo (DOI: 10.5281/zenodo.21916352) and is available without restriction. A statement to this effect has been added to the Data availability section. (The repository DOI, 10.5281/zenodo.21916352, has now been inserted into the Data availability section.)

3. To comply with PLOS One submissions requirements, in your Methods section, please provide additional information regarding the experiments involving animals and ensure you have included details on (1) methods of sacrifice, (2) methods of anesthesia and/or analgesia, and (3) efforts to alleviate suffering.

Response: Thank you. We have added the requested information to the Methods (Mice and housing conditions). The revised text now states the light/dark cycle and husbandry conditions, that marker attachment — the only invasive procedure — was performed under isoflurane anesthesia (1.5–2%), that no analgesia was required because no surgical or otherwise painful procedure was performed, the efforts made to minimize animal number and distress, the daily health monitoring and absence of humane endpoints, and the method of euthanasia at the end of the experimental period.

4. We note that the grant information you provided in the ‘Funding Information’ and ‘Financial Disclosure’ sections do not match.

When you resubmit, please ensure that you provide the correct grant numbers for the awards you received for your study in the ‘Funding Information’ section.

Response: Thank you for noting this discrepancy. The grant numbers listed in the Financial Disclosure are the correct ones, and we will enter these identical numbers in the ‘Funding Information’ section of the online submission form at resubmission so that the two sections match. No change to the manuscript text is required for this point.

5. Thank you for stating the following in the Acknowledgments Section of your manuscript: [We thank the members of the Department of Psychiatry, Nara Medical University, for their technical assistance and support with animal care. This work was supported by The NOVARTIS Foundation for the Promotion of Science, SENSHIN Medical Research Foundation, Mochida Memorial Foundation for Medical and Pharmaceutical Research, Kawano Masanori Memorial Public Interest Incorporated Foundation, Inamori Foundation, Senri Life Science Foundation, Astellas Foundation for Research on Metabolic Disorders, Naito Foundation, and Takeda Medical Research Grants; KAKENHI [Grant-in-Aid for Scientific Research numbers 20K15935 and 24K10684 and Grant-in-Aid for Transformative Research Areas number 21H05698 to KY and JP24K02386 to MM]; the Japan Agency for Medical Research and Development (AMED; grant numbers 22wm0525010h0002 to KY and 24gm1910004h0002, 24gm1510009s0103, 25wm0625510s0102, and JP25wm0625126 to MM); and Moonshot R&D (grant number JPMJMS239F-1-2 to MM). The funders had no role in the study design, data collection, data analysis, data interpretation, writing of the report, or decision to submit the article for publication.]

We note that you have provided funding information that is not currently declared in your Funding Statement. However, funding information should not appear in the Acknowledgments section or other areas of your manuscript. We will only publish funding information present in the Funding Statement section of the online submission form.

Please remove any funding-related text from the manuscript and let us know how you would like to update your Funding Statement. Currently, your Funding Statement reads as follows: “The authors received no specific funding for this work.”

Please include your amended statements within your cover letter; we will change the online submission form on your behalf.

Response: Thank you. All funding-related text has been removed from the Acknowledgements section, which now reads only: “We thank the members of the Department of Psychiatry, Nara Medical University, for their technical assistance and support with animal care.” The complete, amended funding statement is provided in our cover letter for entry into the online submission form on our behalf.

6. In the online submission form, you indicated that [Data are available from the corresponding author upon reasonable request].

All PLOS journals now require all data underlying the findings described in their manuscript to be freely available to other researchers, either 1. In a public repository, 2. Within the manuscript itself, or 3. Uploaded as supplementary information.

This policy applies to all data except where public deposition would breach compliance with the protocol approved by your research ethics board. If your data cannot be made publicly available for ethical or legal reasons (e.g., public availability would compromise patient privacy), please explain your reasons on resubmission and your exemption request will be escalated for approval.

Response: Thank you. We have revised the Data availability section so that the data are made freely available rather than provided on request: the processed behavioral data underlying every figure, together with the underlying positional tracking data, are supplied as Supporting Information (S1 Data), and the analysis code is deposited on Zenodo (DOI: 10.5281/zenodo.21916352). No restrictions apply to the availability of data, code, or materials.

7. Please include your full ethics statement in the ‘Methods’ section of your manuscript file. In your statement, please include the full name of the IRB or ethics committee who approved or waived your study, as well as whether or not you obtained informed written or verbal consent. If consent was waived for your study, please include this information in your statement as well.

Response: Thank you. The full ethics statement has been added to the Methods (Mice and housing conditions): all experimental procedures were approved by the Institutional Animal Care and Use Committee of Nara Medical University (Approval No. 13588) and were conducted in accordance with its guidelines. Because this study involved only animals and no human participants, informed written or verbal consent was not applicable, and no consent waiver was required.

8. If the reviewer comments include a recommendation to cite specific previously published works, please review and evaluate these publications to determine whether they are relevant and should be cited. There is no requirement to cite these works unless the editor has indicated otherwise.

Response: Thank you. We have reviewed the works referred to in the reviewers’ comments and have cited those that we judged directly relevant to the present findings. No citation was added solely because it was mentioned in the review.

Reviewer #1: The report under review “Juvenile social isolation induces long-lasting, sex-specific changes in social behavior and interaction dynamics in mice” examined the impact of post-weaning social isolation on spontaneous social behavior at multiple time points during both the light and dark phases. Overall the study presents some interesting differences across the light cycle that are novel in the field. However, the data presentation and statistical analyses decrease clarity for the reader.

Response: We thank the reviewer for the careful and constructive assessment of our manuscript, and in particular for recognizing the novelty of the light/dark-phase differences. We have revised the statistical reporting, unified the terminology, and clarified the data presentation as detailed below.

1. The statistical analyses in the current manuscript as not entirely appropriate. If there are not significant interactions, then the pair-wise comparisons should not be run. Overall, the large number of comparisons make the take-home message of the findings sometimes difficult to focus on. Perhaps the authors should include a chart with the pairwise comparison data and then focus on the trends across time or within developmental time points. For example, there are developmental trends that are consistent across all groups (i.e. a decrease in social encounter count during the dark phase as they animals get older). Overall, the way the data are presented it is hard to focus on the important differences (i.e. main effects of housing; sex differences and shifts in the developmental time course of these behaviors).

Response: We agree. The Statistical analysis section now states explicitly that pairwise comparisons were performed only for effects for which the corresponding main effect or interaction reached significance in the RM-ANOVA, and that no post hoc test was run in the absence of a significant effect. To reduce the visual burden of the large number of comparisons, the complete pairwise comparison results have been moved to a supporting file (S1 Table), and the figures and Results text now emphasize the main effects of housing condition, sex differences, and the developmental trajectories that are consistent across groups (for example, the decrease in social encounter counts during the dark phase with age). Figures 1–3 have been regenerated and S1 Table has been completed; all values in S1 Table were recomputed directly from the raw data and match the GraphPad Prism output.

2. The lack of consistent and precise language when referring to the different variables decreases clarity as a reader. For example, sometimes activity is called activity and sometimes it is total distance traveled. But then “distance” is sometimes used as shorthand to refer to inter-mouse distance which is a different variable. Another example of this is night is often used in place of dark phase. Dark phase is more precise and should be used throughout the manuscript.

Response: Thank you — this has been corrected throughout. “Night” has been replaced by “dark phase” in every instance in the main text and figure legends; “locomotor activity” is now used consistently for the total distance traveled by an individual mouse; and “inter-mouse distance” is always written in full, with the unqualified term “distance” no longer used for either measure. A sentence defining this terminology has been added to the Methods.

3. The rationale for the discussion of inter-mouse distance along with activity rather than as a measure of social behavior is unclear.

Response: We have added the rationale to the Results. Inter-mouse distance is presented alongside locomotor activity because both are derived from the same continuous positional data and jointly describe how animals distributed themselves in space; it is therefore treated as a spatial-proximity index that is strongly constrained by locomotion, whereas social encounter counts and contact duration serve as the primary measures of social interaction.

4. Are the p-values reported for the post-hoc tests the corrected values based on the multiple comparisons? It is not clear.

Response: Yes. All post hoc p values reported in the text and figures are Bonferroni-corrected values adjusted for the number of multiple comparisons; uncorrected p values are not reported. This has now been stated explicitly in the Statistical analysis section.

5. In some cases the graphs are put on a larger scale to fit in all the comparisons which make it harder to see the data. When there is a main effect of housing condition (i.e. the pairwise comparisons are significant between GH and j-SI in both sexes at all time points – Fig. 1e, 2b) this could be indicated in the figure legend instead of all the individual brackets. Additionally, the figures should clearly indicate not only the figure legend that the red and green dots represent the j-SI groups whereas the GH animals are in gray.

Response: We agree and have revised the figures accordingly: where the pairwise comparisons are significant between GH and j-SI in both sexes at all time points (Fig 1E, Fig 2B), the y-axis was rescaled so that the data are more clearly visible, and the significance of each GH vs. j-SI comparison is annotated directly on the corresponding pair. The colour coding is now stated explicitly in both the figure legend and the figure panels (grey, GH; colour, j-SI: green for males and red for females). During regeneration, every asterisk was re-derived from the raw data; this showed that several Fig 2B p-values in the previous version were inconsistent with the data, and the legend has been corrected accordingly (P49 and P63 males change from *** to ****, and P63 females from * to ***; all comparisons remain significant).

6. Given the much longer time frame of recording during the dark phase (12h vs. 3h), the data for this phase should be broken up into 3h time blocks to allow for more direct comparisons.

Response: Thank you for this suggestion. We would like to clarify the rationale behind our recording design. The 3-h light phase and the 12-h dark phase were never intended as duration-matched windows for direct comparison. The dark phase was the primary observation period because mice are nocturnal and exhibit the great majority of their spontaneous social activity at this time, whereas the short light interval immediately preceding lights-off was included only as an ancillary reference. Consistent with this, the light and dark phases were analyzed entirely separately, and at no point were the two unequal-duration windows compared statistically. Because all of our contrasts are made within a given phase—between housing conditions, sexes, and developmental stages—matching the two phases by subdividing the dark phase into 3-h blocks is not necessary for the comparisons we actually perform. Moreover, adding a time-of-night factor to an already three-factor design (developmental stage × sex × housing) would substantially increase the complexity of the figures and the number of post-hoc comparisons without informing the between-condition questions that are the focus of this study. We have therefore retained the 12-h dark phase as a single analysis window and have clarified this rationale in the Methods.

7. It is not clear from the methods whether the animals began the social interaction test 3hr prior to the end of the light phase such that the light cycle was not shifted during the test. I assume this is the case but it should be stated explicitly. They should also report the lights on/off times and whether the animals are on a 12:12 cycle.

Response: The reviewer’s assumption is correct, and we have now stated this explicitly. The Methods now report that mice were maintained on a 12:12-h light/dark cycle with lights on and off at fixed times, and that recording always began 3 h before lights off, so that the 3-h light phase and the subsequent 12-h dark phase corresponded to the animals’ regular cycle; the light/dark cycle was not shifted at any point during testing.

8. While the entropy measure is clearly defined in the methods, it is not clear whether this construct has been validated and is conveying novel information. The control groups do not demonstrate a developmental shift in this measure for example. The authors should discuss more about what the entropy and switching is telling us that is novel.

Response: Thank you for this important point. We have added a paragraph to the Discussion explaining what entropy and the switching ratio add beyond total contact time and contact frequency — they describe how evenly contacts are distributed across partners and how frequently partners are alternated — and acknowledging explicitly that these measures have not been formally validated against an independent behavioral standard and that GH controls did not show a developmental shift in entropy. We therefore now present them as descriptive indices of partner-selection structure and treat the j-SI group differences as hypothesis-generating.

Reviewer #2: This manuscript investigates the long-term effects of juvenile social isolation on spontaneous social behavior using an automated long-term behavioral monitoring system. The use of longitudinal behavioral assessment in both male and female mice under relatively naturalistic group conditions is an important strength of the study, and the behavioral tracking system is technically impressive.

However, I have several major concerns regarding the experimental design and statistical analysis that substantially limit confidence in the conclusions. In my opinion, these issues cannot be adequately addressed through revision alone.

Response: We thank the reviewer for the positive assessment of the longitudinal design and the tracking platform, and for the detailed critique. We address each concern below and have revised the manuscript accordingly; where a concern requires re-analysis or additional experiments, we state this explicitly rather than overstating what the present revision achieves.

My primary concern is the statistical treatment of the social interaction data. The behavioral measurements (e.g., inter-mouse distance, contact frequency, and contact duration) are generated from interactions among animals within the same cage and are therefore not statistically independent. Nevertheless, the analyses appear to treat pairwise interaction data as independent observations. Because individual animals contribute repeatedly to multiple interaction pairs within the same social group, the effective sample size is substantially inflated, leading to pseudoreplication and potentially exaggerated statistical significance. This issue affects the principal findings throughout the manuscript and would require a fundamentally different analytical framework based on appropriate hierarchical or mixed-effects models.

Response: We agree with the reviewer and thank them for identifying this important issue. Our original analysis did not fully account for the non-independence of pairwise observations within the same four-mouse test group. We therefore reanalyzed all outcomes using linear mixed-effects models with developmental stage, sex, housing condition, and their interactions as fixed effects; random intercepts were included for the four-mouse test group and for the analysis unit (individual mouse or dyad, as appropriate) nested within test group. Thus, the 24 four-mouse groups, rather than the individual pairwise observations, defined the independent clustering level. After accounting for this hierarchy, the developmental stage x sex x housing interaction remained significant for inter-mouse distance during both the light phase (Wald χ²(2) = 28.17, p < 0.001) and dark phase (χ²(2) = 26.45, p < 0.001), and for social encounter count during the dark phase (χ²(2) = 10.66, p = 0.0049) but not the light phase (χ²(2) = 1.28, p = 0.528). The interaction also remained significant for social contact duration during the light phase (χ²(2) = 8.71, p = 0.0128) and dark phase (χ²(2) = 34.63, p < 0.001). We have revised the Statistical analysis and Results sections accordingly and have replaced the previous statement that pairwise observations were not treated as independent. We also note the limited number of independent four-mouse groups as a limitation and have moderated the interpretation of statistical significance.

A second major concern relates to the experimental design itself. Animals remained socially isolated throughout the entire experimental period until the final behavioral assessment. Consequently, the study cannot distinguish persistent effects of juvenile isolation from the ongoing effects of continuous social isolation. Therefore, conclusions regarding the "long-lasting" consequences of juvenile isolation are not fully supported by the experimental design.

Response: We agree that the present design cannot separate the persistent effects of a time-limited juvenile isolation from the effects of continued isolation, because animals remained individually housed until the final assessment. Rather than claim that isolation restricted to the P21–P35 critical window is by itself sufficient, we have reframed the study as a model of continued post-weaning social deprivation and scoped our claims accordingly: “long-lasting” now denotes persistence of the phenotype under ongoing isolation, not irreversibility after resocialization, and we no longer attribute the observed changes to the critical-window manipulation alone. Dissociating the two components would require a resocialization group (isolation restricted to P21–P35 followed by regrouping), as used in prior work, which we identify as the key next experiment. Once the claims are scoped to what a continued-isolation design supports, the interpretation is fully consistent with the data; the Limitations and Discussion have been revised accordingly.

In addition, the comparison between group-housed and isolated animals is confounded by differences in social familiarity. Group-housed mice were tested with continuously familiar cage mates, whereas isolated mice were reunited with littermates only during behavioral testing. This difference represents a potentially important confounding variable for measures of social distance, contact duration, and interaction dynamics, making it difficult to attribute the observed behavioral changes specifically to juvenile social isolation.

Response: We agree that group-housed and isolated animals differ in social familiarity at test. We respectfully note, however, that reduced familiarity is not an extraneous nuisance variable that can be dissociated from isolation: by definition, a socially isolated animal lacks the familiar cage-mates that group housing provides, so diminished familiarity is an intrinsic component of the isolation manipulation rather than a separable confound, and holding familiarity constant would entail removing part of the construct under study. We therefore interpret the reported differences as the combined effect of social deprivation and its inherent reduction in familiarity, and have made this explicit in the Limitations. A design testing group-housed animals with unfamiliar conspecifics could formally partition the familiarity component, and we now note this as an option for future work; we do not think it is required to support the present, appropriately scoped conclusions.

Finally, several interpretations in the Discussion are stronger than supported by the presented data, and some statements are not fully consistent with the reported results.

Response: We have moderated the Discussion accordingly. The interpretations are now presented as provisional, the entropy and switching findings are framed as hypothesis-generating descriptive indices rather than validated constructs, and causal attribution to juvenile isolation is explicitly deferred pending studies with resocialization, familiarity-matched controls, and cage-level statistical modeling.

Overall, although the experimental approach is interesting and the behavioral monitoring platform is valuable, the major concerns regarding statistical independence, experimental design, and interpretation substantially weaken the reliability of the conclusions. Because these issues are fundamental rather than editorial, I cannot recommend publication of the manuscript in its current form.

Response: We are grateful for this frank assessment. We have addressed the statistical-independence concern by performing the cage-clustered mixed-effects reanalysis described above, and we have revised the statistical methods, results, and limitations accordingly. We have also moderated the interpretation and clearly delimited what the present design can and cannot support. The remaining design limitation—distinguishing persistence after resocialization from effects under continued isolation—would require a resocialization control and is now stated explicitly.

Additional editorial corrections

1. The Author contributions statement omitted Natsuko Kashida, duplicated Mamiko Okuda, and used the same initials for two pairs of authors (MT and TY).

Response: We corrected the contribution statement by adding Natsuko Kashida to the experiment and data-analysis contributions, removing the duplicated Mamiko Okuda entries, and spelling out all author names so that Masato Takahashi/Michihiro Toritsuka and Takahiro Yoshihara/Takahira Yamauchi are unambiguous.

2. Several Discussion citations did not support the associated claims, including the citations for hypersocial behavior and the subsequent citation groups.

Response: We rechecked the Discussion citations against the reference list and revised both citations and wording. Hypersocial/increased social-preference findings are now cited to Rivera-Irizarry et al. and Myers et al. [23,24]; aggression to Biro et al. [22]; brief behavioral-assay methodology to the appropriate assay references [25,26,36,37]; long-term group tracking to Endo et al. [28,29]; and circuit, sex-dependent, and developmental claims to the corresponding mechanistic and developmental references. We also corrected the parallel statement in the Introduction.

3. The Abstract overstated the consistency of the inter-mouse-distance effect across developmental stages despite the nonsignificant P63 female light-phase comparison.

Response: We softened the Abstract to state that inter-mouse distance was greater across most developmental stages and sex-phase combinations, which is consistent with Fig 1D and the qualified wording in the Discussion.

Attachment Submitted filename: Response_20260818_revised.docx

# Peer review: Decision Letter 1

**DOI:** 10.1371/journal.pone.0361386.r003

**Authors:** Laurence Coutellier

18 Sep 2026

Dear Dr. Yamamuro,

plosone@plos.org. When you're ready to submit your revision, log on to https://www.editorialmanager.com/pone/ and select the 'Submissions Needing Revision' folder to locate your manuscript file.

- A letter that responds to each point raised by the academic editor and reviewer(s). You should upload this letter as a separate file labeled 'Response to Reviewers'.
- A marked-up copy of your manuscript that highlights changes made to the original version. You should upload this as a separate file labeled 'Revised Manuscript with Track Changes'.
- An unmarked version of your revised paper without tracked changes. You should upload this as a separate file labeled 'Manuscript'.

If you would like to make changes to your financial disclosure, please include your updated statement in your cover letter. Guidelines for resubmitting your figure files are available below the reviewer comments at the end of this letter.

If applicable, we recommend that you deposit your laboratory protocols in protocols.io to enhance the reproducibility of your results. Protocols.io assigns your protocol its own identifier (DOI) so that it can be cited independently in the future. For instructions see: https://journals.plos.org/plosone/s/submission-guidelines#loc-laboratory-protocols. Additionally, PLOS One offers an option for publishing peer-reviewed Lab Protocol articles, which describe protocols hosted on protocols.io. Read more information on sharing protocols at https://plos.org/protocols?utm_medium=editorial-email&utm_source=authorletters&utm_campaign=protocols.

As the corresponding author, your ORCID iD is verified in the submission system and will appear in the published article. PLOS supports the use of ORCID, and we encourage all coauthors to register for an ORCID iD and use it as well. Please encourage your coauthors to verify their ORCID iD within the submission system before final acceptance, as unverified ORCID iDs will not appear in the published article. *Only*  the individual author can complete the verification step; PLOS staff *cannot*  verify ORCID iDs on behalf of authors.

We look forward to receiving your revised manuscript.

Kind regards,

Laurence Coutellier, PhD

Academic Editor

PLOS One

Journal Requirements:

If the reviewer comments include a recommendation to cite specific previously published works, please review and evaluate these publications to determine whether they are relevant and should be cited. There is no requirement to cite these works unless the editor has indicated otherwise.

[Note: HTML markup is below. Please do not edit.]

Reviewers' comments:

Reviewer's Responses to Questions

**Comments to the Author**

Reviewer #3: (No Response)

Reviewer #4: (No Response)

**********

2. Is the manuscript technically sound, and do the data support the conclusions?

Reviewer #3: Partly

Reviewer #4: Yes

**********

3. Has the statistical analysis been performed appropriately and rigorously?

Reviewer #3: No

Reviewer #4: Yes

**********

4. Have the authors made all data underlying the findings in their manuscript fully available?

The PLOS Data policy

Reviewer #3: Yes

Reviewer #4: Yes

**********

5. Is the manuscript presented in an intelligible fashion and written in standard English?

Reviewer #3: Yes

Reviewer #4: Yes

**********

Reviewer #3: While the authors have responded to most comments, two major comments previously raised remain to be clarified, and one statement should be modified unless two new analyses are provided.

1. The authors should explicitly mention in the Abstract and highlight in the relevant sections of the manuscript the most important limitation of their study: isolated mice were tested with unfamiliar mice (cage mates were separated for over two weeks; therefore, they were unfamiliar animals), whereas group-housed mice were tested with their familiar cage mates.

2. The authors should use the statistical approach that is most appropriate for the longitudinal structure of their data. At present, the authors primarily use a three-way repeated-measures ANOVA and subsequently compare these results with those obtained from linear mixed-effects models (LMMs). Given the experimental design, LMM is more appropriate as the data involve repeated and therefore correlated observations within the same animal at P35, P49, and P63, as well as between-subject factors (sex and housing condition). In addition, LMMs provide greater flexibility in handling the unequal numbers of animals across groups, and can account for the hierarchical structure of the data, including potential cage effects, and the different behavioral outcomes may also have different distributions.

Therefore, the authors should use the LMMs as the primary statistical analysis and report the corresponding results consistently throughout the statistical methods, Results, figures, and/or supplementary table. I recommend removing the separate “Cage-clustered reanalyses” section from the Results, or add the RM-ANOVA statistical results and what distinct question it addresses.

In particular, the authors should clarify if the cage effect between the two housing conditions was statistically significant in this model.

3. The statement at Line 432, “From this perspective, our findings do not support the interpretation that isolated males exhibit enhanced sociability,” and similar statements throughout the manuscript appear somewhat too strong given the experimental design and the potential confounding factors.

First, the longitudinal recording approach is a strength of the study and provides an interesting opportunity to characterize social interactions across development. However, the current analysis appears to rely primarily on global parameters aggregated over the recording period. However, the sociability and locomotor activity were not examined as a function of time for each parameter within each session.

This is particularly important because a short-term response to the reintroduction of a partner to isolated animals could provide valuable information. For example, examining the first 5–10 minutes following reunion separately from the remainder of the session could help distinguish an acute response to social re-exposure from overall social interaction across the entire recording period. If such an analysis is done with the existing data, it would substantially strengthen the interpretation. If these additional analyses cannot be performed, the authors should temper their conclusion and specify that their data demonstrate an ‘overall’ reduced sociability under the conditions tested, but could not exclude an acute effect upon reunion, rather than making a broader statement about sociability in isolated males.

Second, aggression/territoriality represents another important potential confounding factor, particularly in isolated male mice. Social isolation alters territorial and aggressive responses in males, and the authors report increased locomotor activity, particularly in males. It is therefore possible that the reduced social interaction observed in isolated males could partly reflect an altered response to the re-exposure of an unfamiliar conspecific, including increased aggression, vigilance, or territorial behavior, rather than only reduced sociability per se. The fact that the social-interaction sessions were repeated at P35, P49, and P63 also makes this issue particularly relevant, as the response to repeated social re-exposure may change over development. The authors should therefore either perform additional analyses for potentially aggressive responses or temper their interpretation and explicitly acknowledge these factors as alternative limitations.

MINOR comments:

4. The use of the term “social behaviors” should be revised throughout the manuscript. The term “social behaviors” is overly broad when the experiments specifically assess sociability/social interaction. Other forms of social behavior were not evaluated in this study (e.g., maternal behavior, territorial behavior, aggression, etc.). I therefore recommend replacing “social behaviors” with more specific terms such as “sociability,” or “social interaction,” depending on the specific term being described.

Reviewer #4: The manuscript titled: ‘Continued post-weaning social isolation is associated with persistent, sex-specific changes in social behavior and interaction dynamics in mice’ by Yamamuro et al. explores the effects of social isolation beginning immediately after weaning on social behavior. This is a well-established topic, but the authors gain a new perspective on the effects of social isolation in several ways. Notable among these are the relatively long period of time for assessing social behavior and the group setting, making the social behavior assay relatively naturalistic. Measurements of behavior including entropy and social switching are also novel and interesting. Sex differences in the effects of isolation are also a bit less studied, so it was nice to have a direct comparison between males and females. As a result, the authors find novel types of effects of social isolation.

In terms of weaknesses, the text of the results would be easier to follow if the statements of statistical outcomes were accompanied by a description of the directions of differences among groups. For example, it doesn’t seem like the text ever states the direction of the sex differences that were discovered, although the existence of sex differences was mentioned multiple times. Whenever posthoc differences are mentioned, it would be helpful to accompany them by saying what those differences are (what direction they are in). This will make it much easier for a reader to follow the results.

The fact that each individual was distinctly marked provides the opportunity to explore the contribution of individual identity to behavior. For example, the lower entropy and switching rate for isolated mice could suggest that there are patterns of association; e.g, that isolated mice spend more time with specific individuals. Is this correct? Is there any indication of whether those individuals are distinct in any way? It doesn’t seem like dominance status was examined, but could factors such as the status of the socially housed individuals matter in patterns of association?

Given the long time period of social interaction, it seems like good opportunity to measure how behavior changes over time. Are the measured behaviors stable over time in the interaction assay or do they change?

Fighting is something that often occurs when isolated mice are placed with conspecifics and is commonly reported to be increased in isolated mice. Did fighting occur with reunification?

Assessing whether mice are approaching versus receiving is described in the methods, but there is no analysis of this in the results. Are there differences between approaching and receiving depending on the housing conditions? This would be a very interesting point relative to other work on social isolation. Additionally, proximity could decrease with affiliation or aggression. Is there a reason to interpret proximity as one or the other?

In general, it would enhance the understanding of and appreciation for the work if the authors could provide more context for some of their findings in the discussion. For example:

- The idea of partner switching and analysis through entropy is interesting and novel; can they explain the concept as it relates to the experimental outcomes more deeply?

- More explanation of what the behavioral differences between light and dark cycles could mean would be interesting, given the novel finding of behavioral differences between light cycles.

It is appropriate to say, as the authors do, that the experiments test the effect of isolation beginning at weaning and extending into adulthood. It is also true that there are several intensive periods of social activity at 35d, 49d, and 63d that could also alter the trajectory of behavior. Could this point be added?

**********

what does this mean?). If published, this will include your full peer review and any attached files.

If you choose “no”, your identity will remain anonymous but your review may still be made public.

**Do you want your identity to be public for this peer review?**  For information about this choice, including consent withdrawal, please see our Privacy Policy

Reviewer #3: **Yes:**  Lucie P. Pellissier

Reviewer #4: No

**********

[NOTE: If reviewer comments were submitted as an attachment file, they will be attached to this email and accessible via the submission site. Please log into your account, locate the manuscript record, and check for the action link "View Attachments". If this link does not appear, there are no attachment files.]

To ensure your figures meet our technical requirements, please review our figure guidelines: https://journals.plos.org/plosone/s/figures

You may also use PLOS’s free figure tool, NAAS, to help you prepare publication quality figures: https://journals.plos.org/plosone/s/figures#loc-tools-for-figure-preparation.

NAAS will assess whether your figures meet our technical requirements by comparing each figure against our figure specifications.

# Author comment: Author response to Decision Letter 2

**DOI:** 10.1371/journal.pone.0361386.r004

25 Sep 2026

Response to Reviewers

Manuscript: “Continued post-weaning social isolation is associated with persistent, sex-specific changes in sociability and social interaction dynamics in mice”

PLOS ONE

We thank the Editor and Reviewers #3 and #4 for their further careful reading and constructive suggestions. We have revised the manuscript accordingly. Below, each comment is reproduced in bold, our response follows in plain text, and text newly added or changed in the manuscript is shown in red. All manuscript changes are marked in red in the revised file; a clean copy is also provided.

Additional corrections made during final verification. While cross-checking the manuscript, supporting tables, and S1 Data, we identified and corrected three data-handling issues; each is documented in S1 Data (README and Analysis_notes). (i) Values that are undefined by construction had been entered as numbers in three light-phase outcomes: switching ratio coded −1 by the source pipeline when fewer than two initiated contacts occurred (n = 18 mouse-stage observations), entropy for mice without an initiated contact (n = 9), and mean contact duration for directed dyads without an initiated encounter (n = 12). All were j-SI observations. They are now treated as missing, consistent with the time-course analysis, and the affected LMMs, contrasts, and figures were recalculated. (ii) Tied highest-count partners (n = 8) are now excluded from the preferred-partner persistence analysis, as stated in the Methods. (iii) Inconsistent labels for four mice at P49 in the switching time-course files were harmonized, and the time-course models were refitted. In addition, in response to Reviewer #3’s request that LMMs be reported consistently, all post hoc comparisons and significance annotations in Figs 1–3 are now LMM contrasts (S2 Table and S1 Data), and RM-ANOVA is reported as complementary omnibus results (S1 Table). Because the complete processed data and all model outputs are provided in S1 Data, the supporting tables were consolidated into three tables: S1 Table (primary LMM and complementary RM-ANOVA results; previously S3 Table), S2 Table (housing and sex contrasts), and S3 Table (exploratory directionality, partner-specificity, and time-course analyses; previously S4–S6 Tables). The consequences for the findings are summarized under Reviewer #3, Comment 2.

Reviewer #3

Comment 1. The authors should explicitly mention in the Abstract and highlight in the relevant sections the most important limitation of their study: isolated mice were tested with unfamiliar mice (cage mates separated for over two weeks; therefore unfamiliar animals), whereas group-housed mice were tested with their familiar cage mates.

Response. We fully agree and thank the reviewer for pressing this point. We had previously described the reunited littermates as “previously familiar,” but we accept that after separation exceeding two weeks—longer than the typical persistence of rodent social recognition memory—the reunited partners should be regarded as effectively unfamiliar. We now state this explicitly in the Abstract, the Methods (Mice and housing conditions), and the Limitations, and we frame reduced familiarity as an intrinsic, inseparable component of the isolation manipulation rather than a nuisance variable.

Abstract (added): “An important design feature is that isolated mice were reunited at testing with cage mates from which they had been separated for more than two weeks and were therefore effectively unfamiliar, whereas group-housed mice were tested with continuously familiar cage mates; the reported effects therefore reflect social isolation together with its intrinsic reduction in social familiarity.”

Methods (revised): “…with whom they had been co-housed only before weaning (until P21). Because these mice had then been separated for more than two weeks before the first test (P35) and for progressively longer before the later tests (P49 and P63)—intervals that exceed the typical duration of rodent social recognition memory—the reunited partners were effectively unfamiliar at testing. In contrast, group-housed mice were tested with cage mates with whom they had remained continuously familiar…”

Limitations (revised, excerpt): “…GH mice interacted with continuously familiar cage mates, whereas j-SI mice were reunited with original littermates after more than two weeks of separation and should be regarded as effectively unfamiliar. Reduced familiarity is therefore inseparable from the isolation manipulation in this design, and the observed differences cannot be attributed to individual housing alone. A familiarity-matched control would require testing both housing groups with unfamiliar conspecifics.”

Comment 2. The authors should use the statistical approach most appropriate for the longitudinal structure of the data. LMM is more appropriate… The authors should use LMMs as the primary statistical analysis and report the results consistently throughout the statistical methods, Results, figures, and/or supplementary table. Remove the separate “Cage-clustered reanalyses” section, or add the RM-ANOVA results and what distinct question it addresses. In particular, clarify if the cage effect between the two housing conditions was statistically significant in this model.

Response. We agree. We recovered the cage and analysis-unit identifiers from the archived source files and recalculated all 12 primary longitudinal models. Each LMM includes developmental stage × sex × housing fixed effects, a random intercept for the four-mouse test group, and a nested random intercept for the analysis unit. The analysis includes 24 independent groups (8 male GH, 5 male j-SI, 6 female GH, 5 female j-SI), 96 mice, 144 undirected dyads, and 288 directed dyads. REML is used for variance components and fixed-effect Wald tests; ML and a boundary-adjusted likelihood-ratio test are used for the cage component. LMMs now provide the primary inference throughout, including all post hoc comparisons: the significance annotations in Figs 1–3, the Results, and the figure legends are based on Bonferroni-adjusted contrasts of LMM estimated marginal means (S2 Table; all contrasts in S1 Data). RM-ANOVA is retained as a complementary within-animal developmental analysis, and its omnibus results are reported in S1 Table. Full results and identifiers are supplied in S1 Table and S1 Data.

Cage effect. The cage random intercept was supported in 11 of 12 models (ML likelihood-ratio χ²(1) = 10.76–129.02, all boundary-adjusted p < 0.001); the exception was light-phase switching ratio after undefined values were treated as missing (χ²(1) = 0.57, p = 0.226). The housing fixed effect was significant in 9 of 12 models; the exceptions were dark-phase contact duration (χ²(1) = 2.44, p = 0.118), light-phase switching ratio (χ²(1) = 0.22, p = 0.639), and dark-phase switching ratio (χ²(1) = 0.15, p = 0.700). Thus, housing-condition differences and residual cage-to-cage heterogeneity are statistically distinct and are now reported separately.

Statistical analysis (revised, excerpt): “…Random intercepts were included for four-mouse test group (cage) and analysis unit nested within cage… Variance components and fixed effects were estimated by restricted maximum likelihood (REML); joint fixed-effect terms were evaluated using Wald χ² tests… The cage random intercept was tested by refitting models by maximum likelihood (ML), comparing the full model with a model omitting cage, and using the 0.5χ²0 + 0.5χ²1 boundary reference…”

For reference, the recalculated stage × sex × housing interactions were: locomotor activity, light χ²(2) = 0.61, p = 0.739 and dark χ²(2) = 9.04, p = 0.0109; inter-mouse distance, light χ²(2) = 27.47, p < 0.001 and dark χ²(2) = 26.73, p < 0.001; social encounter count, light χ²(2) = 1.43, p = 0.488 and dark χ²(2) = 10.51, p = 0.0052; mean contact duration, light χ²(2) = 6.30, p = 0.0429 and dark χ²(2) = 9.67, p = 0.0080; entropy, light χ²(2) = 4.13, p = 0.127 and dark χ²(2) = 6.53, p = 0.0382; switching ratio, light χ²(2) = 2.81, p = 0.245 and dark χ²(2) = 8.63, p = 0.0134.

Consequences of LMM-based contrasts. Because the LMM accounts for cage clustering, several differences that had been significant in the RM-ANOVA post hoc tests were not supported by the LMM contrasts, and the text was revised accordingly. In males, lower switching after j-SI is now confined to the light phase at P35 (previously also reported for the dark phase at P35 and P49), and fewer dark-phase encounters are significant only at P35 (previously P35, P49, and P63). Lower interaction-partner entropy after j-SI is now confined to females (light phase at P35 and P49; dark phase at P49 and P63). Several previously reported sex contrasts (e.g., greater dark-phase locomotor activity in GH males than GH females at P35) were not significant. The central findings—greater inter-mouse distance, later-emerging increases in locomotor activity, reduced early encounter counts, and shorter light-phase contact duration after j-SI—were retained. The Abstract, Results, figure legends, and Discussion were updated.

Comment 3. The statement at Line 432 (“our findings do not support the interpretation that isolated males exhibit enhanced sociability”) and similar statements appear too strong. (a) The data rely on global parameters aggregated over the recording period; sociability was not examined as a function of time within a session. Examining the first 5–10 min after reunion separately from the remainder could distinguish an acute response from overall interaction. (b) Aggression/territoriality is another confound in isolated males. Either perform additional analyses for aggressive responses or temper the interpretation and acknowledge these factors.

Response. We both tempered the interpretation and performed the additional time-course analysis supported by the archived files. Switching ratio was available in six 30-min light-phase bins and twelve 60-min dark-phase bins. It declined within both phases (marginal slopes: light −0.070/h, SE 0.007, p < 0.001; dark −0.0102/h, SE 0.0020, p < 0.001), and the first 30 min of the light phase exceeded the later-bin mean by 0.139 (SE 0.013, p < 0.001). We added these exploratory results, Fig 4, S3 Table, and the time-binned data. The archive does not resolve the first 5–10 min and contains no ethological aggression labels or suitable fine-action video scoring, so those two requested analyses remain impossible from the available data; the manuscript now states this limitation precisely.

Discussion (revised): “…the exploratory switching time course showed higher switching in the first 30 min followed by a within-session decline. This supports an early-versus-later change in interaction sequencing, while the available 30-min bins cannot resolve the first 5–10 min and no comparable time course was available for approach count, duration, locomotion, or aggression.”

Limitations (revised): “…dorsal-marker tracking cannot directly score biting, tail rattling, chasing, wrestling, dominance, or aggression… only switching ratio was available at subphase resolution, in six 30-min light bins and twelve 60-min dark bins. These data demonstrate an early-to-later decline in switching but cannot resolve the first 5–10 min or time courses for other measures…”

Comment 4. (MINOR) The term “social behaviors” is overly broad; replace with more specific terms such as “sociability” or “social interaction” as appropriate.

Response. Done. We have replaced the generic term “social behavior(s)” with “sociability” or “social interaction” throughout, wherever a specific measured construct is meant, retaining the broad term only where we genuinely refer to the general construct (e.g., background statements). We also added a one-sentence operational definition to the Methods.

Methods (added): “Throughout, we use ‘sociability’ to denote the tendency to approach and maintain social contact and ‘social interaction’ to denote the measured contact events and their temporal organization; other categories of social behavior (e.g., aggression, territorial, or maternal behavior) were not assessed.”

For consistency with the measured constructs, we updated the title to “…persistent, sex-specific changes in sociability and social interaction dynamics in mice.”

Reviewer #4

We thank Reviewer #4 for the generous assessment of the novelty and strengths of the study and for a set of suggestions that have substantially improved the clarity and depth of the manuscript.

Comment 4.1 (direction of effects). The results would be easier to follow if statements of statistical outcomes were accompanied by the direction of differences among groups (e.g., the direction of sex differences is never stated); whenever post hoc differences are mentioned, say which direction they are in.

Response. We agree and have added the direction of every reported post hoc difference—housing, sex, and developmental-stage contrasts—in the Results and in all figure legends. Housing and sex contrasts are tabulated with signed estimates in S2 Table, and all contrasts, including developmental-stage contrasts, are provided in S1 Data. For example, within GH mice, males had a shorter light-phase inter-mouse distance than females at P63 and a shorter dark-phase mean contact duration than females at P35; at P63, j-SI females had lower dark-phase entropy than j-SI males; and developmental changes are stated as increases or decreases (e.g., within j-SI mice, distance traveled increased from P35 to P49 and from P49 to P63 in both sexes).

Examples (Results, revised): “j-SI mice had fewer light-phase social encounters than GH mice at P35 in both sexes”; “Light-phase mean contact duration per initiated encounter was shorter in j-SI than in GH females at all three stages”; and “The light-phase switching ratio was lower in j-SI than in GH males at P35 and then increased within j-SI males from P35 to P49 and P63.”

Comment 4.2 (individual identity / partner-specificity). Lower entropy and switching for isolated mice could suggest patterns of association (isolated mice spend more time with specific individuals). Is this correct? Are those individuals distinct in any way? Dominance status was not examined, but could the status of the socially housed individuals matter in patterns of association?

Response. We recovered the light-phase 20-mm directed partner matrices and performed the requested partner-specificity analysis. j-SI mice directed a larger maximum share of initiated events to one partner than GH mice (descriptive means 0.637 vs 0.484; housing Wald χ²(1) = 13.27, p < 0.001), with the same conclusion from the Herfindahl index (0.557 vs 0.396; χ²(1) = 11.15, p < 0.001). We also tracked the identity of the highest-count partner across stages. Persistence was not consistently greater in j-SI mice; all sex-specific Fisher exact comparisons were non-significant (all p ≥ 0.191); mice whose highest count was tied between partners were excluded from this analysis. Thus, isolation was associated with greater concentration on one partner within a session, but not clear developmental persistence of the same partner. Dominance rank and other partner traits were not measured and cannot be inferred. Full results are in S3 Table and S1 Data.

Discussion/Limitations (revised): “Direct partner-level analysis confirmed that j-SI mice concentrated a larger share of initiated light-phase events on a single partner. However, preferred-partner identity was not consistently more stable across development, and the dataset contains no dominance rank or other partner trait with which to characterize the selected individual.”

Comment 4.3 (behavior over time / stability within the assay). Given the long recording period, it seems a good opportunity to measure how behavior changes over time. Are the measured behaviors stable over time in the interaction assay, or do they change?

Response. We agree and have now added the time-course analysis permitted by the archived files. Switching ratio was analyzed over six 30-min light-phase bins and twelve 60-min dark-phase bins, with source values of −1 (undefined because of insufficient interactions) treated as missing. Switching declined within both phases, and the first 30 min was higher than the later light-phase bins. Fig 4, S3 Table, and the full time-binned observations have been added. Because no finer bins or time courses for the other outcomes were present, we do not claim to resolve the first 5–10 min or the stability of every behavioral measure.

Comment 4.4 (fighting on reunification). Fighting often occurs when isolated mice are placed with conspecifics and is commonly increased in isolated mice. Did fighting occur with reunification?

Response. We did not formally score fighting. Because the AR-LABO system records the position of a dorsal ID marker and not fine-scale actions, it cannot detect biting, tail rattling, or wrestling, so we cannot quantify aggression directly and do not wish to over-interpret positional proxies. We now state this limitation explicitly (see also Reviewer #3, point 3b) and note that increased locomotor activity and greater inter-mouse distance in isolated males are compatible with heightened aggression/vigilance but are not diagnostic of it; dedicated video-based ethological scoring will be required.

Comment 4.5 (approach vs. receiving; proximity as affiliation vs. aggression). Approach versus receiving is described in the Methods, but there is no analysis in the Results. Are there differences between approaching and receiving depending on housing? Also, proximity could decrease with affiliation or aggression—is there a reason to interpret proximity as one or the other?

Response. We recovered the light-phase approach and receive records and completed the requested analysis. Each approach by one mouse is the corresponding receive event for its partner, so four-mouse-group totals necessarily balance. The signed individual initiation index showed neither a deviation from zero (p = 0.243) nor a housing effect (p = 0.235). In contrast, the absolute individual imbalance was greater in j-SI mice than GH mice (0.365 vs 0.194; housing χ²(1) = 12.56, p < 0.001), indicating stronger specialization into relatively more initiating or more receiving roles without a population-wide shift in direction. These results are reported in S3 Table and S1 Data. We also clarify that greater spacing is valence-ambiguous and can reflect reduced affiliation, avoidance, vigilance, or territoriality.

Discussion (revised): “Greater inter-mouse distance is interpreted as reduced spatial proximity, but proximity is ambiguous in valence: it may reflect diminished affiliation, active avoidance, vigilance, or territorial spacing toward a less familiar conspecific. Positional data alone cannot distinguish these possibilities.”

Comment 4.6 (more Discussion context: entropy/switching; light vs. dark cycle). Provide more context for findings: explain the concept of partner switching / entropy in relation to the outcomes more deeply, and explain what the behavioral differences between light and dark cycles could mean.

Response. We have expanded the Discussion in both respects.

Discussion (revised, entropy/switching): “…an animal can maintain the same total contact time while distributing it evenly across partners (high entropy) or unevenly (low entropy), and can alternate partners frequently (high switching) or persist with one partner (low switching). The observed reductions therefore indicate a more uneven distribution of contacts and less frequent partner change, not necessarily contact with fewer partners.”

Discussion (added, light vs. dark): “…dark-phase measures reflect behavior during peak activity, when motivational and circadian drives are highest. The stage × sex × housing interactions detected during the dark phase, but not the light phase, for locomotor activity, encounter counts, and switching ratio… are consistent with isolation-related differences being most readily expressed when animals are most active… the phase-dependent pattern suggests that the behavioral consequences of juvenile isolation are state-dependent and most evident during periods of high spontaneous activity.”

Comment 4.7 (intensive social sessions may alter trajectory). It is true that there are several intensive periods of social activity at P35, P49, and P63 that could also alter the trajectory of behavior. Could this point be added?

Response. Yes—this is an important qualification of the longitudinal design, and we have added it.

Discussion/Limitations (added): “…the longitudinal design itself includes three prolonged periods of intensive social interaction (at P35, P49, and P63), during which otherwise isolated mice experienced 15 h of group housing. These repeated re-exposures could themselves influence the developmental trajectory of social behavior—for example, by providing intermittent social experience or by cumulative habituation to the test context—so the later time points reflect not only continued isolation but also the effect of prior testing sessions.”

Data and reproducibility additions. The revised S1 Data workbook now includes the cage/unit identifiers, approach–receive records, partner matrices and selectivity summaries, switching time bins, and all LMM outputs. The analysis code and derived CSV outputs are supplied as a separate reproducibility package, including the reanalysis code for the corrected data, LMM contrasts (new LMM_contrasts and LMM_EMMs sheets), and RM-ANOVA (new RM_ANOVA sheet; S1 Table).

We are grateful to both reviewers; we believe these revisions have materially strengthened the manuscript and hope it is now suitable for publication in PLOS ONE.

Attachment Submitted filename: Response_to_Reviewers_20260926.docx

# Peer review: Decision Letter 2

**DOI:** 10.1371/journal.pone.0361386.r005

**Authors:** Laurence Coutellier

12 Oct 2026

<p>Continued post-weaning social isolation is associated with persistent, sex-specific changes in sociability and social interaction dynamics in mice

PONE-D-26-30469R2

Dear Dr. Yamamuro,

We’re pleased to inform you that your manuscript has been judged scientifically suitable for publication and will be formally accepted for publication once it meets all outstanding technical requirements.

Within one week, you’ll receive an e-mail detailing the required amendments. When these have been addressed, you’ll receive a formal acceptance letter and your manuscript will be scheduled for publication.

An invoice will be generated when your article is formally accepted. Please note, if your institution has a publishing partnership with PLOS and your article meets the relevant criteria, all or part of your publication costs will be covered. Please make sure your user information is up-to-date by logging into Editorial Manager at Editorial Manager® and clicking the ‘Update My Information' link at the top of the page. For questions related to billing, please contact billing support.

If your institution or institutions have a press office, please notify them about your upcoming paper to help maximize its impact. If they’ll be preparing press materials, please inform our press team as soon as possible -- no later than 48 hours after receiving the formal acceptance. Your manuscript will remain under strict press embargo until 2 pm Eastern Time on the date of publication. For more information, please contact onepress@plos.org.

Kind regards,

Laurence Coutellier, PhD

Academic Editor

PLOS One

Additional Editor Comments (optional):

Reviewers' comments:

Reviewer's Responses to Questions

**Comments to the Author**

Reviewer #3: All comments have been addressed

Reviewer #4: All comments have been addressed

**********

2. Is the manuscript technically sound, and do the data support the conclusions?

Reviewer #3: Yes

Reviewer #4: (No Response)

**********

3. Has the statistical analysis been performed appropriately and rigorously?

Reviewer #3: Yes

Reviewer #4: (No Response)

**********

4. Have the authors made all data underlying the findings in their manuscript fully available?

The PLOS Data policy

Reviewer #3: Yes

Reviewer #4: (No Response)

**********

5. Is the manuscript presented in an intelligible fashion and written in standard English?

Reviewer #3: Yes

Reviewer #4: (No Response)

**********

Reviewer #3: (No Response)

Reviewer #4: (No Response)

**********

what does this mean?). If published, this will include your full peer review and any attached files.

If you choose “no”, your identity will remain anonymous but your review may still be made public.

**Do you want your identity to be public for this peer review?**  For information about this choice, including consent withdrawal, please see our Privacy Policy

Reviewer #3: No

Reviewer #4: No

**********

# Editor report: Acceptance letter

**DOI:** 10.1371/journal.pone.0361386.r006

**Authors:** Laurence Coutellier

PONE-D-26-30469R2

PLOS One

Dear Dr. Yamamuro,

I'm pleased to inform you that your manuscript has been deemed suitable for publication in PLOS One. Congratulations! Your manuscript is now being handed over to our production team.

At this stage, our production department will prepare your paper for publication. This includes ensuring the following:

* All references, tables, and figures are properly cited

* All relevant supporting information is included in the manuscript submission,

* There are no issues that prevent the paper from being properly typeset

You will receive further instructions from the production team, including instructions on how to review your proof when it is ready. Please keep in mind that we are working through a large volume of accepted articles, so please give us a few days to review your paper and let you know the next and final steps.

Lastly, if your institution or institutions have a press office, please let them know about your upcoming paper now to help maximize its impact. If they'll be preparing press materials, please inform our press team within the next 48 hours. Your manuscript will remain under strict press embargo until 2 pm Eastern Time on the date of publication. For more information, please contact onepress@plos.org.

You will receive an invoice from PLOS for your publication fee after your manuscript has reached the completed accept phase. If you receive an email requesting payment before acceptance or for any other service, this may be a phishing scheme. Learn how to identify phishing emails and protect your accounts at https://explore.plos.org/phishing.

If we can help with anything else, please email us at customercare@plos.org.

Thank you for submitting your work to PLOS One and supporting open access.

Kind regards,

PLOS One Editorial Office Staff

on behalf of

Dr. Laurence Coutellier

Academic Editor

PLOS One
